# Clinical predictors of pulmonary tuberculosis among South African adults with HIV

**DOI:** 10.1016/j.eclinm.2022.101328

**Published:** 2022-03-05

**Authors:** Simon C. Mendelsohn, Andrew Fiore-Gartland, Denis Awany, Humphrey Mulenga, Stanley Kimbung Mbandi, Michèle Tameris, Gerhard Walzl, Kogieleum Naidoo, Gavin Churchyard, Thomas J. Scriba, Mark Hatherill, the CORTIS-HR Study Team

**Affiliations:** a South African Tuberculosis Vaccine Initiative, Institute of Infectious Disease and Molecular Medicine and Division of Immunology, Department of Pathology, University of Cape Town, Anzio Road, Observatory, Cape Town 7925, South Africa; b Vaccine and Infectious Disease Division, Fred Hutchinson Cancer Research Center, Seattle, WA 98109, USA; c DST/NRF Centre of Excellence for Biomedical TB Research, South African Medical Research Council Centre for TB Research, Division of Molecular Biology and Human Genetics, Department of Biomedical Sciences, Faculty of Medicine and Health Sciences, Stellenbosch University, Cape Town 7505, South Africa; d Centre for the AIDS Programme of Research in South Africa (CAPRISA), Durban 4001, South Africa; e MRC-CAPRISA HIV-TB Pathogenesis and Treatment Research Unit, Doris Duke Medical Research Institute, University of KwaZulu-Natal, Durban 4001, South Africa; f The Aurum Institute, Johannesburg 2194, South Africa; g School of Public Health, University of Witwatersrand, Johannesburg 2193, South Africa; h Department of Medicine, Vanderbilt University, Nashville, TN 37232, USA

**Keywords:** Tuberculosis, Mycobacterium tuberculosis, HIV, Clinical, Model, Prediction, Risk, Diagnosis, Subclinical, Case-finding

## Abstract

**Background:**

Tuberculosis (TB) clinical prediction rules rely on presence of symptoms, however many undiagnosed cases in the community are asymptomatic. This study aimed to explore the utility of clinical factors in predicting TB among people with HIV not seeking care.

**Methods:**

Baseline data were analysed from an observational cohort of ambulant adults with HIV in South Africa. Participants were tested for *Mycobacterium tuberculosis* (Mtb) sensitisation (interferon-γ release assay, IGRA) and microbiologically-confirmed prevalent pulmonary TB disease at baseline, and actively surveilled for incident TB through 15 months. Multivariable LASSO regression with post-selection inference was used to test associations with Mtb sensitisation and TB disease.

**Findings:**

Between March 22, 2017, and May 15, 2018, 861 participants were enrolled; Among 851 participants included in the analysis, 94·5% were asymptomatic and 45·9% sensitised to Mtb. TB prevalence was 2·0% at baseline and incidence 2·3/100 person-years through 15 months follow-up. Study site was associated with baseline Mtb sensitisation (*p* < 0·001), prevalent (*p* < 0·001), and incident TB disease (*p* = 0·037). Independent of site, higher CD4 counts (per 50 cells/mm^3^, aOR 1·48, 95%CI 1·12–1·77, *p* = 0·006) were associated with increased IGRA positivity, and participants without TB disease (aOR 0·80, 95%CI 0·69–0·94, *p* = 0·006) had reduced IGRA positivity; no variables were independently associated with prevalent TB. Mixed ancestry (aHR 1·49, 95%CI 1·30–>1000, *p* = 0·005) and antiretroviral initiation (aHR 1·48, 95%CI 1·01–929·93, *p* = 0·023) were independently associated with incident TB. Models incorporating clinical features alone performed poorly in diagnosing prevalent (AUC 0·65, 95%CI 0·44–0·85) or predicting progression to incident (0·67, 0·46–0·88) TB.

**Interpretation:**

CD4 count and antiretroviral initiation, proxies for immune status and HIV stage, were associated with Mtb sensitisation and TB disease. Inadequate performance of clinical prediction models may reflect predominantly subclinical disease diagnosed in this setting and unmeasured local site factors affecting transmission and progression.

**Funding:**

The CORTIS-HR study was funded by the Bill & Melinda Gates Foundation (OPP1151915) and by the Strategic Health Innovation Partnerships Unit of the South African Medical Research Council with funds received from the South African Department of Science and Technology. The regulatory sponsor was the University of Cape Town.

**Research in context**

**Evidence before this study**

Diagnosis of pulmonary tuberculosis (TB) depends primarily on screening for symptoms consistent with TB and collection of sputum for microbiological testing. However, there is mounting evidence of high prevalence of subclinical (asymptomatic microbiologically-confirmed) TB, that symptom-based case-finding approaches would fail to detect. Effective tools for screening of asymptomatic individuals in communities are urgently needed to inform earlier definitive microbiological testing and treatment, and to interrupt the spread of *Mycobacterium tuberculosis* (Mtb). Medline was searched for studies published in all languages prior to October 1, 2021, that evaluated multivariable clinical prediction models for diagnosing subclinical TB in high burden community settings with terms for “adult”, “clinical”, “prediction”, “model”, and “tuberculosis”. Models identified by the search were predominantly designed for triage of symptomatic individuals seeking care or attending out-patient clinics; algorithms designed for active and passive case-finding were heavily dependent on the presence of symptoms. Only one study tested a clinical risk score for diagnosing subclinical disease in high transmission settings; however, this score had marginal discriminatory performance.

**Added value of this study**

The majority (>80%) of TB cases in this community cohort of people living with HIV were subclinical and would not have been detected by symptom-focused triage algorithms. TB clinical prediction models designed to screen for clinical TB in high-risk populations, such as symptomatic individuals or patients with HIV presenting to clinics, are not suitable for diagnosing subclinical or minimally symptomatic TB in otherwise healthy individuals who are not seeking care. Clinical multivariable models derived in this cohort also performed poorly in diagnosing prevalent TB or predicting progression to incident disease, and did not meet the World Health Organization (WHO) Target Product Profile benchmark criteria for TB triage or prognostic tests. Addition of clinical variables to Mtb-specific T-cell sensitisation (interferon-γ release assay, IGRA) and host-response transcriptomic (RISK11) biomarkers in multivariable models did not improve performance over IGRA or RISK11 alone. Lower CD4 cell counts, indicative of immune suppression, were associated with reduced Mtb sensitisation, consistent with poor performance of IGRA for predicting TB disease. In high TB burden settings, unmeasured local factors affecting Mtb transmission and disease progression likely contributed to poor performance of TB clinical prediction models.

**Implications of all the available evidence**

Clinical prediction models appear inadequate to detect incipient and subclinical TB among people with HIV in community settings. This finding highlights the need for more sensitive TB screening tools to find the “missing” TB cases in high-incidence settings, and to guide targeted confirmatory diagnostic testing and preventive therapy.

Alt-text: Unlabelled box

## Introduction

There were an estimated 209,000 tuberculosis (TB) cases among the 7·5 million people living with HIV (PLHIV) in South Africa in 2019.^1^^,^^2^ Many of these cases go undiagnosed and untreated (treatment coverage 58%) resulting in a case fatality rate of 17%.^2^ Current TB active case-finding strategies rely on symptomatology, however recent TB prevalence surveys in Africa and Asia demonstrate that a large proportion (range 36–80%) of TB cases are asymptomatic,^3^, ^4^, ^5^ including 44% of TB cases in South Africa with a known HIV positive status.^6^ Recent evidence demonstrates aerosolization of *Mycobacterium tuberculosis* (Mtb) among TB patients by tidal breathing, even in the absence of cough;^7^^,^^8^ Sub-clinical TB cases missed by symptom screening may perpetuate Mtb transmission. Novel active case-finding approaches, which do not rely on presence of symptoms, are needed. Chest radiography has been used successfully in prevalence surveys as a screening tool to guide confirmatory sputum microbiological testing and for diagnosis of subclinical TB,^3^, ^4^, ^5^, ^6^ and computer-aided detection offers promise for affordable mass screening.^9^^,^^10^ Rapid point-of-care blood-based tools, such as C-reactive protein (CRP)^11^ and host-blood transcriptomics,^12^ are also currently being investigated.

Simple clinical prediction models are attractive as triage tools to determine need for further investigations for TB suspects or to guide empiric TB preventive therapy and offer a pragmatic solution in resource-limited settings.^13^ Clinical prediction scores incorporating such variables have been designed as triage tools to prioritise investigation for TB among PLHIV at routine clinic visits,^14^ for empiric diagnosis of TB disease in patients presenting to clinics,^15^ and for prognosis of incident TB in low transmission settings.^16^ Most clinical predictive tools are heavily weighted towards TB symptoms,^17^ or are dependent on results of tuberculin skin testing (TST), interferon-γ release assay (IGRA), or radiography.^18^ TST and IGRA positivity reflect Mtb-specific T-cell sensitisation, which is a proxy for prior Mtb infection and is associated with increased risk of TB disease. However, these tests are not able to discriminate between cleared or persistent Mtb infection. Understanding risk factors for Mtb sensitisation may elucidate risk factors associated with prior Mtb infection, and help guide efforts to reduce transmission. No clinical prediction tools have been designed specifically for community screening (active case finding) among TB endemic populations of people living with HIV not seeking care with a large burden of subclinical TB. Such a tool could be employed by community health workers for mass community screening or by nurses at antiretroviral therapy clinic appointments.

Risk factors for TB were studied in a cohort of predominantly ART-experienced South African adults with HIV, who were without clinical suspicion of TB and were not seeking care, and tested whether they were useful for clinical prediction of disease in this context. The association between socio-demographic, clinical, immunological, and virological factors, and baseline risk of Mtb sensitisation (IGRA positivity), prevalent pulmonary TB, and progression to incident pulmonary TB was evaluated using baseline data from a multi-centre prospective observational cohort study.^19^ The diagnostic and prognostic utility of clinical prediction models for pulmonary TB, with and without the addition of IGRA or RISK11, a transcriptomic signature of TB risk,^19^ was then determined and compared with published clinical TB triage tools.

## Methods

This study is reported according to the TRIPOD guidelines.^20^

### Ethics approval

The study protocol was approved by the University of Cape Town Faculty of Health Sciences Human Research Ethics Committee (HREC 812/2017 and 648/2016) and written informed consent was obtained from all participants.

### Study design and participants

Sample size for this sub-analysis was based on enrolment into the CORTIS-HR parent study in PLHIV, which has previously been described.^19^ Briefly, healthy adult volunteers without clinical suspicion of TB, residing in five TB endemic communities in South Africa (Durban, Klerksdorp, Ravensmead, Rustenburg, and Worcester), were recruited through word-of-mouth, house-to-house visits, and liaison with non-governmental organisations. Recruitment did not target symptomatic individuals seeking health care or other high-risk groups. Eligible participants aged 18–59 years were not pregnant or lactating, were without comorbidities (except for HIV), and did not have known TB disease or household exposure to individuals with multi-drug resistant TB within the prior three years. Participants with household exposure to individuals with drug-sensitive TB were eligible.

### Study procedures

Screening procedures included medical history and examination; and HIV rapid antibody test. Enrolment procedures included TB screening and phlebotomy for CD4 cell count, HIV plasma viral load in all ART-naïve participants (post-hoc also in a random subset of ART-experienced participants), IGRA (QuantiFERON TB Gold-Plus, Qiagen, Hilden, Germany), and the RISK11 transcriptomic biomarker. RISK11 was measured as previously described.^21^ Antiretroviral therapy (ART)-naïve participants were referred for ART and isoniazid preventive therapy (IPT) per country guidelines. Participants attended up to seven study visits, including three telephonic contact or field visits at Months 1, 2, and 9; and four site visits at Months 3, 6, 12, and 15 (end of study visit).

### TB screening

Two spontaneously expectorated sputum samples were collected from all enrolled participants who could provide sputum samples at enrolment and at the end of study visits, irrespective of symptoms. End of study visits were performed at month 15 of follow-up or at an earlier timepoint for withdrawn participants. Both samples collected at enrolment and end of study visits were tested for Mtb using Xpert MTB/RIF (enrolment visit; Cepheid, Sunnyvale, CA, USA) or Xpert Ultra (end of study visit; Cepheid) and liquid mycobacterial culture (enrolment and end of study visits; Mycobacteria Growth Indicator Tube [MGIT], BACTEC, Beckton Dickinson, Franklin Lakes, NJ, USA). In addition, symptom-triggered TB investigations (two sputum samples; one for Xpert MTB/RIF and one for MGIT culture) were performed at six routine study visits through 15-months follow-up. TB symptoms included at least one of: persistent unexplained cough, night sweats, fever, or weight loss for 2 weeks or more, or any haemoptysis. Chest radiography was not used in screening for TB. Participants diagnosed with microbiologically-confirmed TB were withdrawn from the study and referred for treatment. Participants diagnosed with TB and/or treated off-study, but who did not meet the study endpoint definition, were also withdrawn.

### Study endpoints

The Mtb sensitisation endpoint was defined as a positive baseline IGRA ≥0·35 IU/mL at study enrolment. The microbiologically-confirmed pulmonary TB disease endpoint was defined by a positive Xpert MTB/RIF, Ultra, and/or MGIT culture on at least one sputum sample. All Xpert Ultra trace positive results were excluded from analysis due to chance of false positivity, and results were considered negative for Mtb. Participants with a TB endpoint within 30 days of the enrolment visit were classified as prevalent TB cases. Collection of sputum samples at baseline irrespective of symptoms was intended to “wash out” all subclinical prevalent TB cases, thus any new cases occurring 30 days after enrolment were considered incident. Participants with a TB endpoint from the end of month 1 through month 15 of follow-up were defined as incident TB cases. Individuals who remained TB free until study discontinuation, or end of follow up at the month 15 end of study visit were classified as TB negative.

### Regression modelling

To identify baseline risk factors of Mtb sensitisation, prevalent TB, and incident TB among cohort participants, both univariable regression and multivariable regularised least absolute shrinkage and selection operator (LASSO) regression analyses were performed. Participants with any randomly missing data points, due to missed data or failed sample collection, were excluded from analyses. HIV plasma viral load was excluded from multivariable regression as it was only measured in a subset of participants. Although imputation is possible with a high rate of missingness, it is not advisable when data are not missing at random and may be dependent on observed data. The Likelihood-ratio test was used to test for significance of each categorical variable (such as study site) in the univariate analysis. A logistic regression modelling approach was used for Mtb sensitisation and prevalent TB endpoints, and a Cox Proportional-Hazards approach was employed for incident TB; Odds and hazard ratios, respectively, were obtained by exponentiating the regression coefficients. Post-selection inference^22^ to compute *p*-values and 95% confidence intervals for the LASSO estimates based on the fixed value of lambda was performed using the *selectiveInference* R package.^23^ Variables with *p* < 0·05 are considered to have a significant association with the outcome. The LASSO regression modelling methodology is detailed in the **Supplementary Methods**.

### Validation of published clinical TB prediction rules

The literature was searched for published diagnostic or prognostic clinical TB prediction rules which were not wholly contingent on the presence of TB symptoms.^17^ It was not possible to validate several scoring systems^18^^,^^24^, ^25^, ^26^, ^27^ due to variables that were not included in the dataset, such as chest radiograph or TST results. There were three tools that were measurable using the clinical variables collected in the CORTIS-HR study: Hanifa2017^14^ was designed to prioritise investigation for TB among PLHIV at routine clinic visits, Baik2020^15^ designed for empiric diagnosis of TB disease in patients presenting to clinics, and Periskope-TB^16^ designed for prediction of incident TB in low transmission settings. Computations of these clinical prediction scores are detailed in the referenced papers and summarised in the **Supplementary Methods**. Diagnostic and prognostic performance of published clinical prediction tools was assessed with receiver operating characteristic (ROC) analysis and area under the curve (AUC) with 95% confidence intervals calculated with a non-parametric percentile bootstrap with 10,000 resamples.

### Training and testing of new TB prediction models

To determine the performance of clinical prediction models for pulmonary TB derived in this cohort, diagnostic and prognostic TB disease risk models using LASSO penalisation for feature selection were constructed with (A) clinical variables alone, (B) IGRA or RISK11 in combination with clinical variables, and (C) IGRA or RISK11 alone. Study site was excluded from these models due to lack of generalisability. The cohort was divided into a training set (67%), to derive the diagnostic and prognostic clinical prediction models, and the remaining test set (33%), in which performance was tested (i.e. 3-fold cross-validation). As few TB cases were observed, this process was repeated 1000 times to obtain more reliable cross-validated area under the ROC curve (CV-AUC) estimates and 95% confidence intervals.

### Role of the funding sources

Subject-specific experts at the Bill & Melinda Gates Foundation and South African Medical Research Council contributed to scientific discussions relating to CORTIS-HR protocol development and study design. The funders of the study had no role in the collection, analysis, and interpretation of data; in the writing of the report; and in the decision to submit the paper for publication.

## Results

### Recruitment of a prospective community cohort of people living with HIV (PLHIV)

Between March 22, 2017, and May 15, 2018, 861 adults living with HIV were enrolled in the CORTIS-HR study at five geographically diverse sites in South Africa. 851 (98·8%) participants were included in this sub-analysis; ten participants with missing IGRA result (*n* = 7), CD4 cell count (*n* = 2), or body-mass index (BMI) measurement (*n* = 1) were excluded (**Supplementary Figure 1**). Participants had a median CD4 cell count of 530·0 (IQR 353·5–726·0) and 77·7% (661/851) were on ART (**Supplementary Table 1**). Participant characteristics varied considerably by site (**Supplementary Table 2)**. 45·9% (391/851; 95%CI 42·6–49·3) participants were sensitised to Mtb (IGRA positive) and there were 17 microbiologically-confirmed prevalent pulmonary TB cases (prevalence 2·0%, 95%CI 1·3–3·2) with one or more positive sputum samples, 88·2% (15/17) were asymptomatic at diagnosis. Amongst the 829 participants with no prevalent TB at baseline, and who attended at least one subsequent visit, 690 (83·2%) participants completed 15-months follow-up per protocol and remained TB disease-free; 21 (2·3 per 100 person-years, 95%CI 1·3–3·3) participants progressed to microbiologically-confirmed incident pulmonary TB disease, 42·9% (9/21) were asymptomatic at diagnosis; and 118 (14·2%) did not complete the study, because of withdrawal, death, or loss to follow-up.

### Immunosuppression (CD4 cell count), TB disease status, and study site were strongly associated with prevalence of Mtb sensitisation

Risk factors associated with Mtb sensitisation at enrolment amongst 391 IGRA positive and 460 IGRA negative participants were explored (**Supplementary Table 1**). Smoking was found to be associated with increased risk of Mtb sensitisation in the univariable analysis (odds ratio [OR] 1·71, 95%CI 1·30–2·26, *p* < 0·001), but the confidence interval bounds included 1 in the multivariable analysis (adjusted odds ratio [aOR] 1·19, 95%CI 0·96–2·97, *p* = 0·040). In the univariable analysis, study site was strongly associated (Likelihood ratio test *p* < 0·001) with Mtb sensitisation. Using LASSO analysis to select variables and regularise a sparse binomial regression model, and selective inference to interpret the results, study site remained a significant co-variable, with increased Mtb sensitisation risk at the Klerksdorp site as compared to all other sites (aOR 1·27, 95%CI 1·00–1·49, *p* = 0·024). After adjusting for co-variables, there was a 1·48-fold (95%CI 1·12–1·77, *p* = 0·006) increase in adjusted odds of Mtb sensitisation for every 50 cell/mm^3^ increase in CD4 cell count. Unsurprisingly, participants without prevalent TB and who did not subsequently progress to incident TB were less likely (aOR 0·80, 95%CI 0·69–0·94, *p* = 0·006) to be Mtb sensitised. No other variables were significant after adjusting for covariates. Similar associations were observed when removing site from the LASSO model in a sensitivity analysis (**Supplementary Table 3**).

### Study site was significantly associated with prevalence of TB disease

Elevated HIV viral load and immunosuppression were found to be associated with prevalence of pulmonary TB disease at baseline in the univariable logistic analysis, with 8·21-fold (95%CI 1·55–151·41, *p* = 0·046) increased unadjusted odds in those with detectable plasma virus and lower odds for every 50 cell/mm^3^ increase in CD4 cell count (OR 0·89, 95%CI 0·80–0·98, *p* = 0·028), (Table 1); higher BMI was also associated with reduced odds for every kg/m^2^ increase (OR 0·90, 95%CI 0·81–0·98, *p* = 0·028). Study site was also significantly associated with prevalent TB disease in the univariable analysis (Likelihood ratio test *p* < 0·001) and was the only significant variable in the selective inference analysis, with 4·59-fold (95%CI 1·49–10·74, *p* = 0·006) increased adjusted odds in participants from Durban, KwaZulu-Natal compared to other sites.

**Table 1:** Regression analyses examining factors associated with prevalent TB disease in people living with HIV.

	No prevalent TB, *n* (%) or median (IQR) *N* = 834	Prevalent TB, *n* (%) or median (IQR) *N* = 17	Univariable logistic regression		Multivariable binomial LASSO regression with selective inference	
			OR (95%CI)	*p* value	aOR (95%CI)	*p* value
Sex						
Female	608 (72·9)	9 (52·9)	Reference		Reference	
Male	226 (27·1)	8 (47·1)	2·39 (0·89, 6·33)	0·076	1·34 (0·08, 2·88)	0·58
Age	35 (29–42)	36 (33–42)	1·02 (0·97, 1·08)	0·39	1·29 (0·00, 2·17)	0·77
Ethnicity						
Black African	704 (84·4)	12 (70·6)	Reference		–	–
Mixed Ancestry	130 (15·6)	5 (29·4)	2·26 (0·71, 6·19)	0·13		
Site				**<0·001**^**‡**^		
Durban, KwaZulu-Natal	288 (34·5)	7 (41·2)	Reference		4·59 (1·49, 10·74) ^†^	**0·006**
Klerksdorp, North West	160 (19·2)	0 (0·0)	NA	NA	–	–
Rustenburg, North West	158 (18·9)	0 (0·0)	NA	NA	–	–
Ravensmead, Western Cape	89 (10·7)	0 (0·0)	NA	NA	–	–
Worcester, Western Cape	139 (16·7)	10 (58·8)	2·96 (1·11, 8·31)	**0·031**	5·08 (0·67, 10·63) ^†^	0·051
Highest level of schooling						
Primary school or lower	100 (12·0)	0 (0·0)	NA		Reference	
Secondary school or higher	734 (88·0)	17 (100·0)		NA	2·29 (0·54, 7·66)	0·11
Employment						
Employed	131 (15·7)	3 (17·6)	Reference		–	–
Unemployed	703 (84·3)	14 (82·4)	0·87 (0·28, 3·81)	0·83		
Occupants per household	4 (3–6)	4 (1–6)	0·87 (0·69, 1·04)	0·17	0·83 (0·27, 26·81)	0·65
Smoking history						
No	513 (61·5)	9 (52·9)	Reference		Reference	
Yes	321 (38·5)	8 (47·1)	1·42 (0·53, 3·75)	0·47	0·60 (0·31, 17·78)	0·52
Prior TB						
No	630 (75·5)	13 (76·5)	Reference		Reference	
Yes	204 (24·5)	4 (23·5)	0·95 (0·27, 2·72)	0·93	0·75 (0·50, 78·17)	0·73
TB household contacts						
No	674 (80·8)	17 (100·0)	NA		Reference	
Yes	160 (19·2)	0 (0·0)		NA	0·35 (0·13, 1·61)	0·076
Isoniazid preventive therapy at enrolment						
Not on therapy	788 (94·5)	17 (100·0)	NA		–	–
On therapy	46 (5·5)	0 (0·0)		NA		
Antiretroviral therapy at enrolment				0·21^‡^		
Naïve	183 (21·9)	7 (41·2)	Reference		1·13 (0·03, 1·73) ^†^	0·77
<6 months	112 (13·4)	2 (11·8)	0·47 (0·07, 1·97)	0·35	–	–
≥6 months	539 (64·6)	8 (47·1)	0·39 (0·14, 1·12)	0·071	–	–
Body-mass index (kg/m^2^)	24·3 (20·6–31·3)	21·3 (18·7–24·0)	0·90 (0·81, 0·98)	**0·028**	0·33 (0·10, 1·67)	0·078
CD4-positive cell count (cells/mm^3^)	530·5 (354·2–731·0)	393·0 (248·0–591·0)	0·89 (0·80, 0·98) *	**0·028**	0·62 (0·20, 1·71) *	0·15
HIV plasma viral load						
<100 copies/mL	179 (45·1)	1 (9·1)	Reference		^#^	^#^
≥100 copies/mL	218 (54·9)	10 (90·9)	8·21 (1·55, 151·41)	**0·046**		
Not tested (missing)	437	6	NA	NA		
Season at enrolment				0·17^‡^		
Spring	196 (23·5)	8 (47·1)	Reference		1·41 (0·66, 196·86) ^†^	0·081
Summer	218 (26·1)	2 (11·8)	0·22 (0·03, 0·91)	0·061	–	–
Autumn	188 (22·5)	3 (17·6)	0·39 (0·08, 1·37)	0·17	–	–
Winter	232 (27·8)	4 (23·5)	0·42 (0·11, 1·36)	0·16	0·85 (0·58, >1000) ^†^	0·88
TB symptoms at enrolment						
No	789 (94·6)	15 (88·2)	Reference		Reference	
Yes	45 (5·4)	2 (11·8)	2·34 (0·36, 8·63)	0·27	1·18 (0·04, 2·11)	0·72
IGRA status						
Negative	455 (54·6)	5 (29·4)	Reference		Reference	
Positive	379 (45·4)	12 (70·6)	2·88 (1·06, 9·12)	**0·049**	1·63 (0·62, 3·20)	0·14

IQR, interquartile range. CI, confidence interval. OR, odds ratio. aOR, adjusted OR. IGRA, interferon-γ release assay. TB, tuberculosis. NA, not applicable. *Per 50 cells/mm^3^. ^#^Excluded from LASSO analysis. ^†^Reference group includes all other levels of variable. ^‡^Likelihood-ratio test for significance of categorical variable.

HIV plasma viral load was excluded from the LASSO regression due to missing data (443/851; 52·1%), and CD4 cell count (*p* = 0·15) and BMI (*p* = 0·078) was not a significant predictor in the multivariable analysis. When site was removed from the LASSO model in a sensitivity analysis, there was a higher prevalence of TB among participants recruited in Spring (1·47, 95%CI 1·23– >1000, *p* = 0·014) (**Supplementary Table 4**). IGRA status (*p* = 0·038), CD4 cell count (*p* = 0·040), and prior household exposure to an individual with TB (*p* = 0·035) were also associated with prevalent TB in this sensitivity analysis, but the 95% confidence interval included 1.

### Ethnicity and initiation of antiretroviral therapy were associated with higher rate of incident TB

Detectable baseline HIV viral load was associated with a 3·69-fold (95%CI 1·04–13·10, *p* = 0·043) increased hazard of progression to incident TB, while higher CD4 cell count (per 50 cell/mm^3^ increase, hazard ratio [HR] 0·86, 95%CI 0·78–0·95, *p* = 0·003) and BMI (per kg/m^2^, HR 0·89, 95%CI 0·81–0·97; *p* = 0·007) were associated with lower risk of progression in the univariable Cox proportional hazards analyses (Table 2).

**Table 2:** Regression analyses examining baseline factors associated with progression to incident TB disease within 15 months in people living with HIV.

	No incident TB, *n* (%) or median (IQR) *N* = 808	Incident TB, *n* (%) or median (IQR) *N* = 21	Univariable Cox proportional hazards		Multivariable Cox LASSO regression with selective inference	
			HR (95%CI)	*p* value	aHR (95%CI)	*p* value
Sex						
Female	590 (73·0)	15 (71·4)	Reference		–	–
Male	218 (27·0)	6 (28·6)	1·11 (0·43, 2·85)	0·83		
Age	35 (29–42)	33 (30–40)	0·98 (0·93, 1·03)	0·41	0·67 (0·44, 8·16)	0·51
Ethnicity						
Black African	689 (85·3)	11 (52·4)	Reference		Reference	
Mixed Ancestry	119 (14·7)	10 (47·6)	5·50 (2·34, 13·00)	**<0·001**	1·49 (1·30, >1000)	**0·005**
Site				**0·037^‡^**		
Durban, KwaZulu-Natal	281 (34·8)	6 (28·6)	Reference		–	–
Klerksdorp, North West	158 (19·6)	1 (4·8)	0·33 (0·04, 2·75)	0·31	0·52 (0·00, 2·20) ^†^	0·061
Rustenburg, North West	154 (19·1)	2 (9·5)	0·68 (0·14, 3·39)	0·64	–	–
Ravensmead, Western Cape	84 (10·4)	5 (23·8)	3·36 (1·03, 11·00)	**0·045**	–	–
Worcester, Western Cape	131 (16·2)	7 (33·3)	2·42 (0·81, 7·20)	0·11	0·99 (0·00, 1·10) ^†^	0·97
Highest level of schooling						
Primary school or lower	95 (11·8)	5 (23·8)	Reference		Reference	
Secondary school or higher	713 (88·2)	16 (76·2)	0·44 (0·16, 1·21)	0·11	0·87 (0·49, 62·58)	0·78
Employment						
Employed	130 (16·1)	1 (4·8)	Reference		Reference	
Unemployed	678 (83·9)	20 (95·2)	3·59 (0·48, 26·80)	0·21	1·80 (0·53, 3·14)	0·18
Occupants per household	4 (3–6)	4 (3–8)	1·09 (0·97, 1·23)	0·15	1·15 (0·00, 1·67)	0·85
Smoking history						
No	501 (62·0)	9 (42·9)	Reference		–	–
Yes	307 (38·0)	12 (57·1)	2·23 (0·94, 5·30)	0·069		
Prior TB						
No	614 (76·0)	12 (57·1)	Reference		Reference	
Yes	194 (24·0)	9 (42·9)	2·24 (0·94, 5·31)	0·068	1·61 (0·73, 5·15)	0·093
TB household contacts						
No	654 (80·9)	16 (76·2)	Reference		–	–
Yes	154 (19·1)	5 (23·8)	1·30 (0·48, 3·56)	0·60		
Isoniazid preventive therapy during study conduct						
Did not receive therapy	409 (50·6)	13 (61·9)	Reference		Reference	
Received therapy	399 (49·4)	8 (38·1)	0·56 (0·23, 1·36)	0·20	0·67 (0·00, 24·08)	0·13
Antiretroviral therapy (ART) during study conduct				0·065^‡^		
ART experienced	635 (78·6)	12 (57·1)	Reference		–	–
ART naïve	42 (5·2)	1 (4·8)	1·84 (0·24, 14·20)	0·56	–	–
ART started after enrolment	131 (16·2)	8 (38·1)	3·08 (1·26, 7·52)	**0·014**	1·48 (1·01, 929·93) ^†^	**0·023**
Body-mass index (kg/m^2^)	24·5 (20·7–31·5)	19·9 (18·5–24·9)	0·89 (0·81, 0·97)	**0·007**	0·56 (0·39, 142·36)	0·68
CD4-positive cell count (cells/mm^3^)	533·5 (360·8–734·2)	291·0 (145·0–573·0)	0·86 (0·78, 0·95) *	**0·003**	0·45 (0·29, 8·85) *	0·37
HIV plasma viral load						
<100 copies/mL	175 (46·1)	3 (20·0)	Reference		^#^	^#^
≥100 copies/mL	205 (53·9)	12 (80·0)	3·69 (1·04, 13·10)	**0·043**		
Not tested (missing)	428	6	–	–		
Season at enrolment				0·10^‡^		
Spring	190 (23·5)	6 (28·6)	Reference		–	–
Summer	210 (26·0)	5 (23·8)	0·70 (0·21, 2·30)	0·56	–	–
Autumn	187 (23·1)	1 (4·8)	0·16 (0·02, 1·34)	0·091	0·45 (0·01, 1·24) ^†^	**0·048**
Winter	221 (27·4)	9 (42·9)	1·18 (0·42, 3·30)	0·76	–	–
TB symptoms at enrolment						
No	764 (94·6)	20 (95·2)	Reference		–	–
Yes	44 (5·4)	1 (4·8)	1·01 (0·14, 7·50)	>0·99		
IGRA status						
Negative	446 (55·2)	7 (33·3)	Reference		Reference	
Positive	362 (44·8)	14 (66·7)	2·43 (0·98, 6·01)	0·056	1·48 (0·60, 10·69)	0·13

IQR, interquartile range. CI, confidence interval. HR, hazard ratio. aHR, adjusted HR. IGRA, interferon-γ release assay. TB, tuberculosis. *Per 50 cells/mm^3^. ^#^Excluded from LASSO analysis. ^†^Reference group includes all other levels of variable. ^‡^Likelihood-ratio test for significance of categorical variable.

In the multivariable LASSO regression, starting ART after study enrolment was weakly associated with increased risk (adjusted hazard ratio [aHR] 1·48, 95%CI 1·01–929·93, *p* = 0·023) of incident TB as compared to ART-naïve participants who did not start ART and to those already on ART at baseline, possibly due to immune reconstitution inflammatory syndrome (IRIS). Self-reported mixed ancestry (aHR 1·49, 95%CI 1·30–>1000, *p* = 0·005) was the only other variable significantly associated with increased progression to incident TB disease in the inferences from the multivariable Cox regression. Although study site was associated with incident TB in the univariable analysis (Likelihood ratio test *p* = 0·037), no sites were significant in the LASSO regression. With study site removed from the model, mixed ancestry (aHR 1·56, 95%CI 1·22–209·44; *p* = 0·004) was the only factor associated with incident TB (**Supplementary Table 5**).

As an exploratory analysis, prevalent and incident TB cases were combined in a binomial LASSO regression model to increase power for selective inference. Lower CD4 cell count (per 50 cell/mm^3^ increase, aOR 0·51, 95%CI 0·34–0·81, *p* = 0·004), lower BMI (per kg/m^2^, aOR 0·47, 95%CI 0·28–0·78, *p* = 0·003), and IGRA positivity (aOR 1·58, 95%CI 1·01–2·59, *p* = 0·023) were found to be independently associated with cumulative TB disease (**Supplementary Table 6**).

### Published clinical prediction rules perform poorly for active case-finding in asymptomatic populations

The study enrolled ambulant PLHIV who were not actively seeking care and were predominantly asymptomatic (804/851; 94·5%); Symptom screening had poor sensitivity (11·8%, 95%CI 0·0–30·0) with a lower than expected number of symptomatic TB cases (2/17).

The diagnostic and prognostic performance of three clinical prediction tools were compared with the RISK11 transcriptomic signature and IGRA, as measured in the parent CORTIS-HR study; 39 participants missing RISK11 scores were excluded to allow for equitable comparison of predictive tools, with resultant exclusion of one prevalent and one incident TB case (**Supplementary Figure 1**).

**Figure 1:**
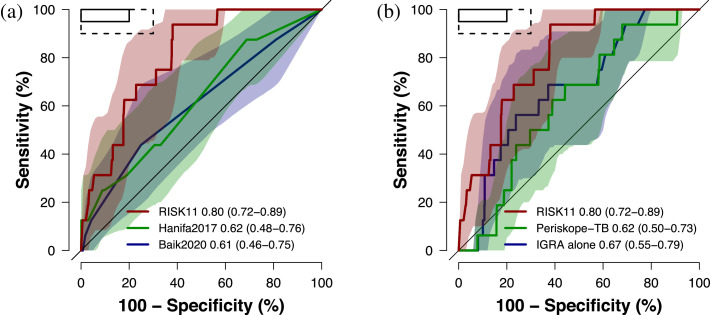
Diagnostic performance of published clinical prediction rules versus the RISK11 transcriptomic signature Receiver operating characteristic (ROC) curves comparing diagnostic performance (area under the curve, AUC, with 95% CI) for prevalent TB diagnosed on one or more liquid culture-positive or Xpert MTB/RIF-positive sputum samples of (a) the RISK11 transcriptomic signature versus the Hanifa2017 and Baik2020 clinical prediction scores, and (b) the RISK11 transcriptomic signature versus the Periskope-TB tool (intended for prognosis in low TB incidence settings) and interferon-γ release assay (IGRA). The shaded areas represent the 95% CIs. The solid box depicts the optimal criteria (95% sensitivity and 80% specificity) and the dashed box depicts the minimal criteria (90% sensitivity and 70% specificity) set out in the WHO Target Product Profile for a TB triage test.^28^

**Figure 2:**
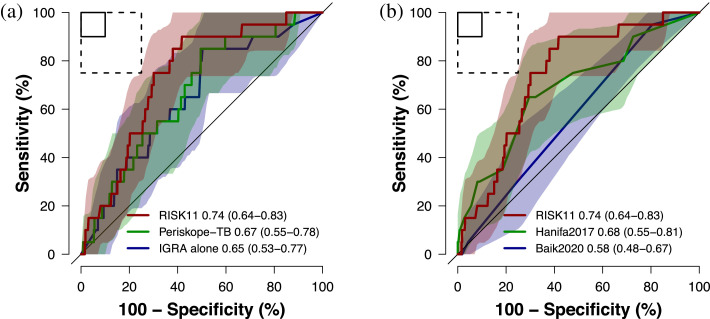
Prognostic performance of published clinical prediction rules for incident TB through 15 months follow-up versus the interferon-γ release assay and RISK11 transcriptomic signature The receiver operating characteristic (ROC) curves comparing prognostic performance (area under the curve, AUC, with 95% CI) for incident TB diagnosed on one or more liquid culture-positive or Xpert MTB/RIF-positive sputum samples through 15 months of follow-up of (a) the RISK11 transcriptomic signature versus the Periskope-TB prognostic tool and interferon-γ release assay (IGRA), and (b) the RISK11 transcriptomic signature versus the Hanifa2017 and Baik2020 clinical prediction scores (intended for diagnosis). The shaded areas represent the 95% CIs. The solid box depicts the optimal criteria (90% sensitivity and 90% specificity) and the dashed box depicts the minimal criteria (75% sensitivity and 75% specificity) set out in the WHO Target Product Profile for an incipient TB test.^29^

The Hanifa2017 and Baik2020 tools performed poorly for diagnosis of prevalent pulmonary TB, with AUCs of 0·62 (95%CI 0·48–0·76) and 0·61 (95%CI 0·46–0·75), respectively, both inferior to RISK11 (*p* = 0·002 and *p* = 0·025; Figure 1**a**). IGRA and Periskope-TB were also inferior to RISK11 for diagnosing prevalent TB (*p* = 0·035 and *p* = 0·002, respectively; Figure 1**b**). None of the clinical prediction rules met the minimum World Health Organization (WHO) Target Product Profile (TPP) performance benchmark criteria for a TB triage test (90% sensitivity and 70% specificity) .^28^ The Periskope-TB tool was modified for use in this high incidence setting for prognosis of incident TB through 15 months follow-up (AUC 0·67, 95%CI 0·55–0·78) and it was not superior to IGRA alone (AUC 0·65, 95%CI 0·53–0·77; *p* = 0·49; Figure 2**a**). RISK11 had a higher AUC (0·74, 95%CI 0·64–0·83), and approached the minimum WHO TPP performance criteria for a TB prognostic test (75% sensitivity and 75% specificity) ,^29^ but was not significantly better than Periskope-TB (*p* = 0·28). When applied as a prognostic test, the Hanifa2017 clinical prediction rule showed equivalent performance to Periskope-TB (*p* = 0·67; Figure 2**b**).

### Derivation of new TB models, or combination of clinical factors with host biomarkers, failed to improve performance over existing tools

The results raised the question whether it is possible to predict TB using clinical and demographic variables in such a setting. Models incorporating clinical features alone, with study site excluded, performed poorly in diagnosing prevalent TB (CV-AUC 0·65, 95%CI 0·44–0·85; **SupplementaryFig. 2**) or predicting progression to incident TB through 15 months follow-up (CV-AUC 0·67, 95%CI 0·46–0·88; **SupplementaryFig. 3**). Household TB exposure history (96·7%), education level (95·6%), CD4 cell count (93·6%), and sex (90·1%) were the most frequent variables included in prevalent TB models; ethnicity (97·5%) and CD4 cell count (92·9%) were most frequently included in incident TB models. Incorporating RISK11 or IGRA into the diagnostic or prognostic LASSO models did not improve performance over RISK11 or IGRA alone, and no model met the minimum WHO TPP performance benchmarks (**Supplementary Figure. 4,5**).

## Discussion

TB clinical prediction models were tested for active case-finding in a community setting of ambulant PLHIV. These models were mainly designed for passive case-finding, or for screening for advanced clinical TB. It is thus not surprising that they performed poorly in a population of mostly asymptomatic adults with HIV who were not seeking care. Derivation of new clinical multivariable models in this cohort or synthesis of clinical data with existing Mtb-specific T-cell sensitisation and host-response transcriptomic biomarkers, IGRA and RISK11, did not improve diagnostic or prognostic discrimination for predominantly subclinical TB.

In keeping with the literature,^30^ lower CD4 cell counts were associated with reduced IGRA positivity, likely due to loss or dysfunction of Mtb-specific T-cell memory responses associated with HIV, and may explain poor prognostic performance of the QuantiFERON TB Gold-Plus assay in this setting. Despite this, it is however noteworthy that IGRA positivity was independently associated with cumulative TB disease in this study. The observed associations between HIV viraemia, immunosuppression (lower CD4 cell count), ART initiation (with resultant recovery of the CD4 cell compartment and possible immune reconstitution inflammatory syndrome), and lower BMI, with greater risk of TB disease are well described.^31^, ^32^, ^33^, ^34^, ^35^, ^36^ In this study, site had an association with both Mtb sensitisation and TB disease. This finding may reflect unmeasured local transmission and progression risk factors, such as undiagnosed co-morbid disease prevalence (e.g. diabetes or alcoholism), differences in the environment, and inherent population susceptibility (i.e. genetic traits).^37^, ^38^, ^39^ This inference is supported by the association of self-reported ethnicity with incident TB, although mixed ancestry may be a proxy variable for the Western Cape Province sites (Worcester and Ravensmead), where people of mixed ancestry comprise a large proportion of the population.^40^ Local transmission dynamics are likely responsible for site level differences in Mtb sensitisation. TB incidence across South Africa is highly heterogenous, ranging from 419 to 880 per 100,000 in Bojanala (Rustenburg site) and Cape Winelands (Worcester site) districts, respectively.^41^ Despite efforts to standardise study procedures across sites, the possibility cannot be excluded that there were operational disparities in investigation for Mtb sensitisation and TB disease between the participating sites, including recruitment methods, clinical history taking, or method of sputum sample collection. It is acknowledged that the effect of study site on Mtb infection and TB disease risk may limit the generalisability of the findings to settings beyond South Africa. High community Mtb exposure with stochastic probability of infection may partly account for poor predictive utility of clinical prediction tools in these settings. Study site is often not acknowledged as an important predictor in the TB clinical prediction modelling literature. For a model to be generalisable between settings, the variables included in the model should predict TB independently of site, or site factors (e.g. local TB prevalence) should be included in the model. Our results demonstrate that site is one of the most significant predictors of TB disease, and are illustrative of the lack of generalisability and poor validation of TB clinical prediction models between different settings.

This study was conducted in community settings with high prevalence of undiagnosed subclinical TB. These results may be pertinent to other TB-HIV co-endemic communities, however they may not be generalisable to case-finding settings characterised by advanced or symptomatic TB, such as household or close contacts, and people seeking care. Despite high median CD4 cell count and high prevalence of ART in this cohort, reflecting a generally healthy population, CD4 cell count remained an important predictor of both incident and prevalent TB in the clinical prediction models. This is pertinent as South Africa approaches 70% ART coverage among PLHIV, similar to the 78% coverage observed in this study.^1^ It is possible that other unmeasured laboratory tests reflecting chronic inflammation, such as monocyte-lymphocyte ratio^42^ and CRP,^11^ might also perform well in this setting. A major strength of this study was the collection of sputum for Xpert and culture testing from all participants who could provide samples, facilitating TB diagnosis early in the disease course. Subclinical and mildly symptomatic TB diagnosed in this study likely reflect a subtle disease phenotype, which may require more sensitive screening tools to detect, such as a transcriptomic signature.^43^^,^^44^ This suggestion is supported by the finding that the RISK11 incipient TB signature outperformed IGRA and the clinical prediction models. Several studies have shown that combining biomarkers with clinical risk factors may improve prediction of TB disease or treatment outcomes.^16^^,^^26^^,^^45^, ^46^, ^47^, ^48^, ^49^ In our endemic community setting, combining clinical variables with host biomarkers (RISK11 and IGRA) did not improve predictive performance for prevalent or incident TB. However, it might be possible to train a more discriminatory model if a larger number of subclinical TB cases were available.^16^^,^^50^ Chest radiography with computer-aided detection has reasonable performance as a rule-out test for symptomatic individuals with presumptive TB^9^^,^^10^ and presents a feasible and affordable tool which is already purposed for active case-finding, however sensitivity for subclinical TB has yet to be ascertained.

In addition to limited generalisability beyond South Africa, this study has other limitations. Despite intensive community active case-finding, sputum sample collection from all enrolled participants who could provide sputum samples at enrolment and end-of-study visits, and symptom-triggered investigation during follow-up, there were few cumulative TB cases limiting power and reducing precision of estimates. Few cases of extra-pulmonary TB were expected in this relatively healthy cohort and therefore, participants were not investigated for disease at other anatomical sites, potentially missing those cases. Chest radiography was also not performed. The low TB case accrual is however an accurate reflection of the true population prevalence and incidence of TB, compared to an artificially inflated, but more balanced, case-control sample. A substudy of a prospective cohort was used and thus no a-priori sample size calculation was carried out. Given the wide and overlapping confidence intervals for the clinical prediction models, results should be interpreted cautiously.

The study findings highlight that clinical prediction scores developed for use in high-risk populations, or for case-finding amongst symptomatic individuals seeking care, may fare poorly for diagnosing predominantly subclinical TB amongst generally healthy people not seeking care in a community setting. While using clinical predictors in combination with biomarkers is a pragmatic, cost-effective alternative, these data suggest that in a community (active case-finding) setting, developing new highly sensitive diagnostic tools is warranted to detect sporadic early subclinical, minimally symptomatic, or incipient TB disease. The role of basic clinical and laboratory variables, such as BMI and CD4 cell count, combined with host-response biomarkers to enhance diagnostic and treatment response performance for TB disease, is yet to be elucidated.

## Contributors

SCM, TJS, and MH conceived the study. MT, GW, KN, and GC were responsible for all site-level activities, including recruitment, clinical management, and data collection. SCM, AF-G, HM, and SKM verified the underlying data. AF-G, DA, HM, and SKM provided statistical support. SCM analysed data. SCM, TJS, and MH interpreted results and wrote the first draft of the manuscript. All authors had full access to the data, and reviewed, revised, and approved the manuscript before submission. MH and TJS had final responsibility for the decision to submit for publication.

## Data sharing statement

Deidentified individual participant clinical metadata, TB endpoint data, and data dictionary are available on Zivahub (https://doi.org/10.25375/uct.14176484), an open access data repository hosted by the University of Cape Town's institutional data repository powered by Figshare for Institutions.

## Funding

The CORTIS-HR study was funded by the Bill & Melinda Gates Foundation (OPP1151915) and by the Strategic Health Innovation Partnerships Unit of the South African Medical Research Council (SAMRC) with funds received from the South African Department of Science and Technology. The regulatory sponsor was the University of Cape Town.

## Declaration of interests

AFG, GW, GC, TJS, and MH report grants from the Bill & Melinda Gates Foundation (BMGF), during the conduct of the study. GW and TJS report grants from the South African Medical Research Council (SA-MRC), during the conduct of the study. GC reports a subcontract from the University of Cape Town to the Aurum Institute. GW reports a subcontract from the University of Cape Town [BMGF OPP1116632] to Stellenbosch University; grants from the National Institutes of Health and The European and Developing Countries Clinical Trials Partnership; patents pending or issued for “TB diagnostic markers” [PCT/IB2013/054377 (USA)], “Serum host biomarkers for tuberculosis disease” [PCT/IB2017/052142], and “Method for diagnosing tuberculosis” [AP/P/2016/009427 (ARIPO), 201580023042.X (China), 15755681.2 (Europe), 201617030869 (India), F/P/2016/258 (Nigeria), 2016/06324 (South Africa)]; and participation on an Independent Data Monitoring Committee [Gates MRI-TBV02-202 Study: NCT04556981] and a Safety Advisory Board [TB Sequel Study: NCT03251196]. MH reports institutional clinical trial grants to the University of Cape Town. TJS reports a patent pending for the RISK11 signature. All other authors declare no competing interests.

## Supplementary materials

Image, application 1
