# Supplementary material for: Clinical predictors of pulmonary tuberculosis among South African adults with HIV
Source: eClinicalMedicine. 2022 Mar 5;45:101328. doi: 10.1016/j.eclinm.2022.101328 (PMC8902614; doi:10.1016/j.eclinm.2022.101328)
Supplement: Supplementary file 1 [file mmc1.pdf]

Supplement to:

Clinical predictors of pulmonary tuberculosis among South African adults with HIV

Simon C. Mendelsohn, Andrew Fiore-Gartland, Denis Awany, Humphrey Mulenga, Stanley Kimbung Mbandi, Michèle Tameris, Gerhard Walzl, Kogieleum Naidoo, Gavin Churchyard, Thomas J. Scriba, Mark Hatherill, and the CORTIS-HR Study Team.

Contents

The CORTIS-HR Study Team 2
Supplementary Methods 4
Supplementary Figures 6
Supplementary Tables 10
References 16

The CORTIS-HR Study Team

Centre for the AIDS Programme of Research in South Africa (CAPRISA), and [7]MRC-CAPRISA HIV-TB Pathogenesis and Treatment Research Unit, Doris Duke Medical Research Institute, University of KwaZulu-Natal, Durban, South Africa.
Bianca Bande
Thilagavathy Chinappa
Cara-mia Corris
Thobelani Cwele
Celaphiwe Dlamini
Dhineshree Govender
Goodness Gumede
Zanele Gwamada
Senzo Halti
Zandile Jali
Lungile Khanyile
Jabu Maphanga
Nonhle Maphanga
Razia Hassan-Moosa
Nompumelelo Ngcobo
Gloria Ntanjana
Nesri Padayatchi
Dirhona Ramjit
Thandiwe Shezi
Chandrapharbha Singh
Phindile Sing
Philile Thembela
Londiwe Zaca
Mbali Zulu

DST/NRF Centre of Excellence for Biomedical TB Research and SAMRC Centre for TB Research, Division of Molecular Biology and Human Genetics, Department of Biomedical Sciences, Faculty of Medicine and Health Sciences, Stellenbosch University, Cape Town, South Africa.
Petri Ahlers
Roslyn Beukes
Maria Didloff
Marika Flinn
Bernadine Fransman
Andriëtte Hiemstra
Jaftha Kruger
Stephanus T. Malherbe
Onesisa Mpofu
Mpho Mtlali
Dorothy Solomons
Kim Stanley
Susanne Tönsing
Khayalethu Toto
Ayanda Tsamane

South African Tuberculosis Vaccine Initiative, Institute of Infectious Disease and Molecular Medicine, Division of Immunology, Department of Pathology, University of Cape Town, South Africa.
Charmaine Abrahams
Hadn Africa
Denis Arendsen
Nomfuneko Cynthia Batyi
Nicole Bilek
Samentra Braaf
Sivuyile Buhlungu
Alida Carstens
Yolundi Cloete
Lorraine Coetzee
Alessandro Companie
Ilse Davids
Marwou de Kock
Bongani Diamond
Palesa Dolo
Mzwandile Erasmus
Juanita Ferreira
Christal Ferus
Michelle Fisher
Hennie Geldenhuys
Diann Gempies
Yolande Gregg
Katie Hadley
Rieyaat Hassiem
Roxane Herling
Yulandi Herselman
Chris Hikuam
Henry Issel
Lungisa Jaxa
Ruwiyda Jansen
Fabio Julies
Fazlin Kafaar
Masooda Kaskar
Sophie Keffers
Xoliswa Kelepu
Gloria Khomba
Sandra Kruger
Sunelza Lakay
Thelma Leopeng
Angelique Kany Kany Luabeya
Simbarashe Mabwe
Lebohang Makhethe
Faheema Meyer
Miriam Moses
Boitumelo Mosito
Angelique Mouton
Munyaradzi Musvosvi
Julia Noble
Onke Nombida
Fajwa Opperman
Adam Penn-Nicholson
Christel Petersen
Patiswa Plaatjie
Abe Pretorius
Rodney Raphela
Frances Ratangee
Maigan Ratangee
Elisma Schoeman
Constance Schreuder
Alison September
Cashwin September
Justin Shenje
Marcia Steyn
Sonia Stryers
Liticia Swanepoel
Anne Swarts
Asma Toefy
Petrus Tyambetyu
Linda van der Merwe
Elma van Rooyen
Habibullah Valley
Ashley Veldsman
Helen Veldtsman
Kelvin Vollenhoven
Elaine Zimri

TB Modelling Group, TB Centre, Centre for Mathematical Modelling of Infectious Diseases, Department of Infectious Disease Epidemiology, London School of Hygiene & Tropical Medicine, London, United Kingdom.
Tom Sumner
Richard G. White

The Aurum Institute, Johannesburg, Gauteng, South Africa.
Aurum Klerksdorp Site
Tebogo Badimo
Kagiso Baepanye
Kesenogile Edna Baepanye
Ken Clarke
Marelize Collignon
Audrey Lebohang Dhlamini
Candice Eyre
Tebogo Feni
Welseh Phindile Galane
Thelma Goliath
Craig Innes
Bonita Janse Van Rensburg
Olebogeng Jonkane
Boitumelo Sophy Kekana
Gomotsegang Virginia Khobedi
Marietjie King
Ndlela Israel Kunene
Aneesa Lakhi
Nondumiso Langa
Hildah Ledwaba
Immaculate Mabasa
Tshegofatso Dorah Mabe
Nkosinathi Charles Mabuza

Molly Majola
Mantai Makhetha
Vernon Malay
Juanita Market
Lungile Mbata
Karabo Moche
Joseph Panie Moloko
Kabelo Molosi
Primrose Mothaga
Nhlamulo Ndlovu
Bantubonke Bertrum Ntamo
Tedrius Ntshauba
Thandiwe Papalagae
Pearl Nomsa Sanyaka
Letlhogonolo Seabela
Raesibe Agnes Pearl Selepe
Melissa Neo Senne
Moeti Serake
Mugwena Thompo
Vincent Tshikovhi
Lebogang Isaac Tswaile

Aurum Rustenberg Site
Laudicia Tshenolo Bontsi
Obakeng Peter Booi
Mari Cathrin Botha
William Brumskine
Kgomotso Violet Chauke
Mooketsi Theophillius Cwaile
Isabella Johanna Davies
Emilia De Klerk
Blanchard Mbay Iyemosolo
James Michael Jeleni
Christian Mabika Kasongo
Christian Mabika Kasongo
Sebaetseng Jeanette Kekana
Lucky Sipho Khoza
Gloria Keitumetse Kolobe
Lerato Julia Lekagane
Sheiley Christina Lekotloane
Ilze Jeanette Louw
Sarah Teboso Lusale
Perfect Tiisetso Maatjie
Kamogelo Fortunate Mabena
Johanna Thapelo Madikwe
Octavia Mahkosazana Madikwe
Rapontwana Letlhogonolo Maebana
Malobisa Sylvester Magwasha
Vutlhari-I-Vunhenha Fairlord Manzini
Isholedi Samuel Maroele
Omphile Petunia Masibi
July Rocky Mathabanzini
Tendamudzimu Ivan Mathode
Ellen Ditaba Matsane
Lungile Mbata
Nyasha Karen Mhandire
Thembisiwe Miga
Nosisa Charity Thandeka Mkhize
Caroline Mkhokho
Neo Hilda Mkwalase
Nondzakazi Mnqonywa
Brenda Matshidiso Modisaotsile
Patricia Pakiso Mokgetsengoane
Selemeng Matseliso Carol Mokone
Kegomoditswe Magdeline Molatlhegi
Thuso Andrew Molefe
Motlatsi Evelyn Molotsi
Tebogo Edwin Montwedi
Boikanyo Dinah Monyemangene
Hellen Mokopi Mooketsi
Tshplpfelo Mapula Mosito
Ireen Lesebang Mosweu
Banyana Olga Motlagomang
Funeka Nomvula Mthembu
Themba Phakathi
Mapule Ozma Phatshwane
Victor Kgothatso Rameetse
Kelebogile Magdeline Segaetsho
Ni Ni Sein
Melissa Neo Senne
Sifiso Cornelius Shezi
Zona Sithetho
Bongiwe Stofile
Mando Mmakhora Thaba
Lethabo Collen Theko
Dimakatso Sylvia Tsagae

Vaccine and Infectious Disease Division, Fred Hutchinson Cancer Research Center, Seattle, WA, USA.
Bhavesh Borate
Eva Chung
Michelle Chung
Alicia Sato
Ellis Hughes

Supplementary Methods

Statistical analysis

All analyses were performed in R (Boston, MA, USA), version 3.6.1.

Analysis cohorts

All enrolled participants were used to evaluate associations with baseline *Mycobacterium tuberculosis* (Mtb) sensitisation and prevalent pulmonary tuberculosis (TB). All participants who did not meet the baseline prevalent TB endpoint, and who attended at least one follow-up visit, were included in evaluation of baseline predictors of incident pulmonary TB. Participants who discontinued follow-up prior to 15 months follow-up and did not meet the incident TB study endpoint definition were censored at their final study visit or last negative sputum sample collection. For determination of the diagnostic and prognostic performance of clinical prediction models for pulmonary TB, only enrolled participants with available interferon-γ release assay (IGRA) and RISK11 results were included.

Approach to missing data

Variables considered for inclusion in the analyses were captured in the CORTIS-HR study with low missingness (<10%), had existing evidence of association with TB disease, and were likely to be available to healthcare practitioners in resource-constrained settings. Missing data points, due for example to failed sample collection, were considered missing completely at random, and these participants were excluded from analyses (i.e. complete case analysis only).

Prevalence estimates

The prevalence of Mtb sensitisation or TB disease (%) was estimated as the proportion of participants with positive baseline IGRA or TB at enrolment. The 95% confidence intervals on prevalence estimates was calculated using the Binomial Wilson method using the R *binom* package.[1]

TB incidence

The incidence (per 100 person-years) of TB through 15 months was estimated among participants using a time-dependent right-censored non-parametric approach (estimate and variance derived from the Nelson-Aalen estimator[2] of cumulative hazard). Participants with prevalent TB at enrolment, or who only attended the initial enrolment visit (follow-up time = 0), were excluded from this estimate.

Least absolute shrinkage and selection operator regression analyses

Least absolute shrinkage and selection operator (LASSO) regression analyses, built using the *glmnet* R package[3], were used to select variables to obtain a parsimonious model and improve prediction accuracy and interpretability. Variables are excluded by LASSO regression (coefficient shrunk to zero) if they are collinear or they do not have an association with the outcome variable, or have very small coefficients and are therefore unlikely to have an association with the outcome variable. To obtain the optimal value of the tuning parameter (lambda), 500 iterations of five-fold cross-validation were performed using a range of lambda values. Balanced folds (i.e. supervised assignment of participants to folds according to dependent/outcome variables) were used to achieve proportional numbers of endpoints in each fold. A choice of five folds was made to yield error rates that do not suffer from either very high variance or high bias.[4] The mean binomial deviance for each fixed value of lambda across the 500 iterations of cross validation were computed, and lambda with the minimum binomial deviance was selected.

Baik2020 clinical score computation

The Baik et al. (*PloS Medicine*, 2020) clinical score (Baik2020) was designed for empiric diagnosis of TB among adults presenting to primary health clinics while awaiting microbiological results, and was calculated as per the published methods[5]:

**Predictor**		**Score**
**Sex**	Female	0
	Male	1
**Age**	<25 or >44 years	0
	25-44 years	1
**HIV status**	Negative	0
	Positive	2
**Diabetes**	Non-diabetic	0
	Diabetic	1
**Symptom duration > 14 days**	No	0
	Yes	1
**Number of WHO TB symptoms reported**	One point each for cough (1), fever (1), night sweats (1), and loss of weight (1)	0-4
		/10

Hanifa2017 clinical score computation

The Hanifa et al. (*PloS One*, 2017) clinical score (Hanifa2017) was designed as a diagnostic prediction model for symptomatic adults attending clinic for routine HIV care to prioritise TB investigation, and was calculated as per the published methods[6]:

**Predictor**		**Score**
**ART status**	On ART ≥3 months	0
	Pre-ART or ART <3 months	3
**BMI, kg/m^2**	≥25	0
	18.5-24.9	2
	<18.5	6
**CD4 cell count, cells/mm^3**	≥350	0
	200-349	1
	<200	3
**Number of WHO TB symptoms reported**	0-1 symptom	0
	>1 symptom	4
		/16

Personalized risk predictor for incident TB (Periskope-TB) computation

The Personalized Risk Predictor for Incident TB (Periskope-TB) clinical prediction tool (Gupta et al., *Nature Medicine* 2020) combines a quantitative measure of T cell sensitisation (IGRA or tuberculin skin test result) and clinical covariates to predict incident TB in low transmission settings (annual incidence ≤20 per 100,000 persons) with minimal risk of reinfection.[7] The R code provided by the authors (github.com/rishi-k-gupta/PERISKOPE-TB) was used to calculate Periskope-TB scores in our cohort, with the following variables and assumptions:

**Variable**	**Assumption**
Age (years)	
Maximum QuantiFERON TB antigen response (higher of TB1 and TB2 tubes; IU/mL)	
QuantiFERON negative control (IU/mL)	
Was the person tested through contact tracing? (Yes/No)	Participants with known household contacts: Yes<br>Participants without known household contacts: No
Proximity of index case (Household/non-household)	Household
Sputum smear status of index case (Positive/Negative)	Positive
Was the person tested born abroad? (Yes/No)	Yes
Country of birth	South Africa
Approximate date of migration	Set to date of enrolment
Is the person tested known to be living with HIV? (Yes/No)	
Has the person tested received a solid organ or haematological transplant? (Yes/No)	
Preventative treatment commenced? (Yes/No)	
Date of latent TB test	

Performance of prediction models, RISK11, and IGRA

Diagnostic and prognostic performance was assessed with binary receiver operating characteristic (ROC) analysis to calculate the AUC using the *pROC* R package[8]. Binary ROC AUCs were compared using the DeLong test.[9] Sensitivity, specificity, positive predictive value (PPV), and negative predictive value (NPV) at each threshold were calculated using binary endpoint indicators and standard formulae. The 95% confidence intervals (CIs) on diagnostic and prognostic performance estimates were calculated with a non-parametric percentile bootstrap with 10,000 resamples.[10] Diagnostic and prognostic accuracy were benchmarked against the minimum WHO target product profile (TPP) thresholds for a community-based TB triage or referral test[11] (specificity 70% and sensitivity 90%) and the minimal WHO TPP thresholds for an incipient TB test, to predict progression to TB disease[12] (specificity 75% and sensitivity 75%), respectively.

Derivation and testing of LASSO clinical prediction models in the CORTIS-HR cohort

For the derivation and testing of diagnostic and prognostic TB clinical prediction models, the cohort was randomly split into training (67%) and test (33%) sets with proportional numbers of TB cases in each (i.e. 3-fold cross-validation). The training set was used to construct diagnostic and prognostic TB disease risk models using LASSO penalisation for feature selection, with or without the addition of RISK11 or IGRA. Study site was not included in the LASSO clinical prediction models as this variable is not generalisable to other settings.

Binomial LASSO regression analyses were built using the *glmnet* R package[3]. To obtain the optimal value of the tuning parameter (lambda), 50 iterations of five-fold cross-validation were performed using a range of lambda values. Balanced folds (i.e. supervised assignment of participants to folds according to dependent/outcome variables) were used to achieve proportional numbers of endpoints in each fold. The mean area under the curve (AUC) for each fixed value of lambda across the 50 iterations of cross validation was computed, and lambda with the maximum AUC across the 50 cross-validation iterations selected. Model performance was then evaluated in the test set. This procedure was repeated 1,000 times to obtain more reliable AUC estimates, randomly splitting the cohort into 1,000 training and test sets to derive and test LASSO models. The mean cross-validated AUC (CV-AUC) estimates and 95% confidence interval across the 1,000 iterations are reported. Relative feature importance was calculated as the number of times each feature was included in a model dived by the total iterations of model training.

Supplementary Figures

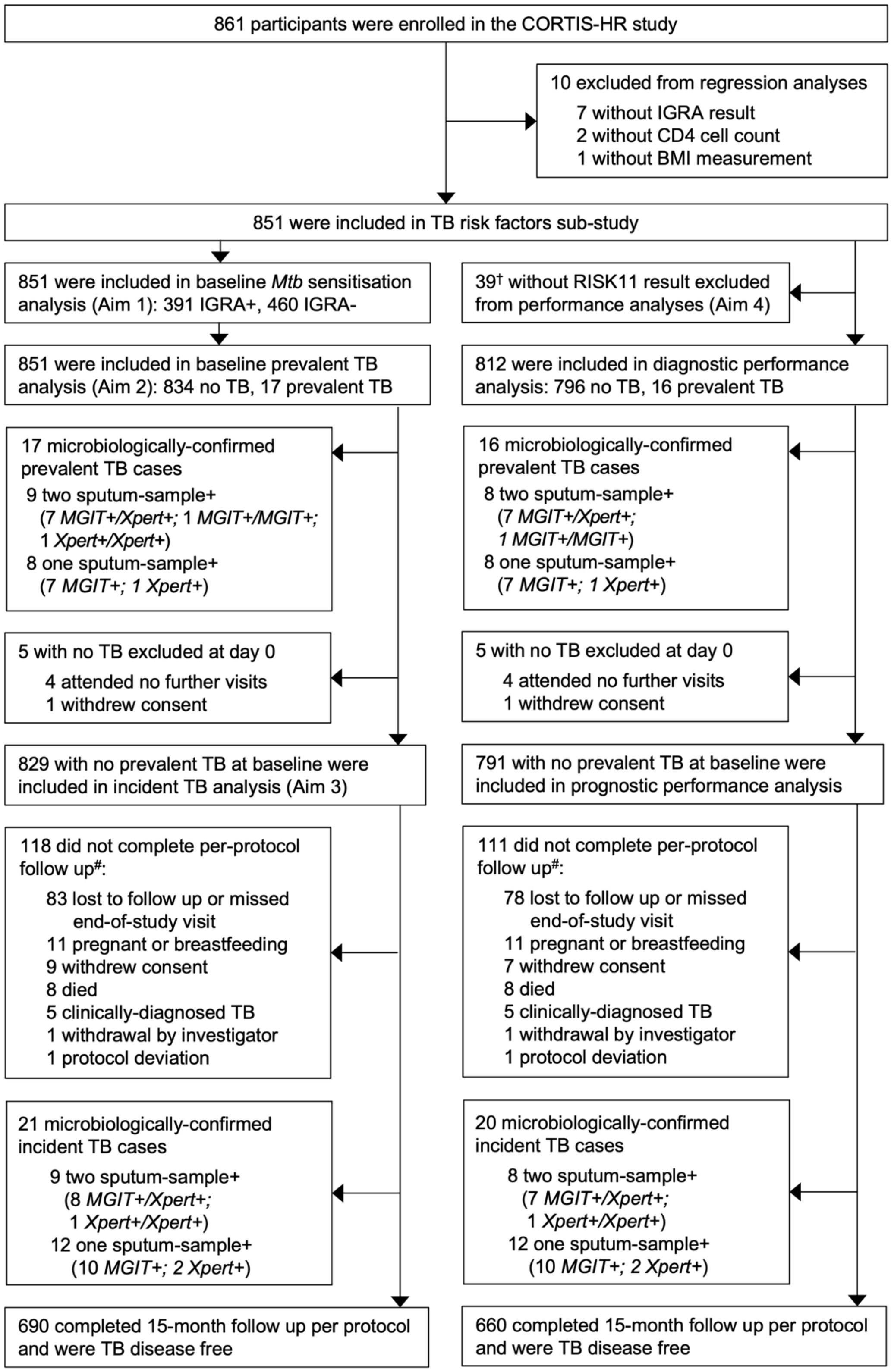

Supplementary Figure 1: Enrolment and follow-up

#Participants who did not complete follow-up per-protocol were included in incident TB analysis but censored as TB disease free at their last study visit. Eight deaths were reported during study follow-up; three of unnatural causes, one of acute gastroenteritis, three of unknown causes, and one from suspected abdominal TB.

†Two without PAXgene RNA and 37 with indeterminate result due to inadequate quality of the RNA sample.

TB, tuberculosis (disease). *Mtb*, *Mycobacterium tuberculosis*. IGRA, interferon-γ release assay. BMI, body-mass index. MGIT, Mycobacteria Growth Indicator Tube. Xpert, GeneXpert MTB/RIF or Ultra.

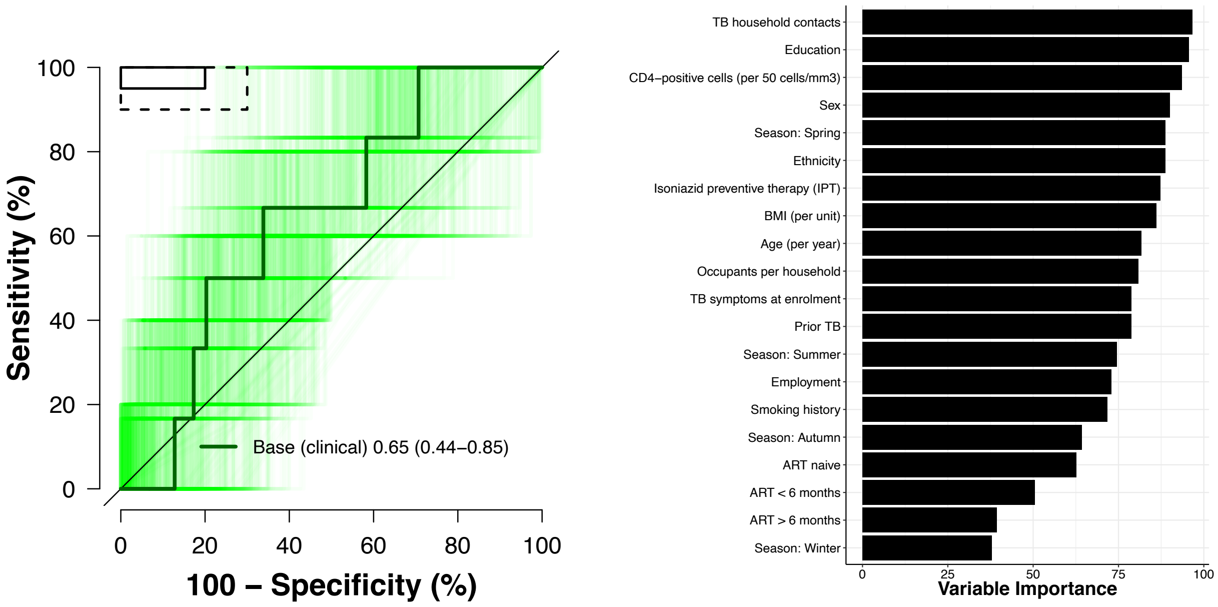

Supplementary Figure 2: Diagnostic performance of LASSO regression clinical prediction models derived and tested in the CORTIS-HR cohort

(a) The receiver operating characteristic (ROC) curves depict test set diagnostic performance (area under the curve, AUC, with 95% CI) of 1,000 LASSO regression clinical prediction models for prevalent TB diagnosed on one or more liquid culture-positive or Xpert MTB/RIF-positive sputum samples. Each of the 1,000 green ROC curves depict performance in an individual test set. The mean cross-validated AUC is represented in dark green. The solid box depicts the optimal criteria (95% sensitivity and 80% specificity) and the dashed box depicts the minimal criteria (90% sensitivity and 70% specificity) set out in the WHO Target Product Profile for a TB triage test.[11] (b) Relative feature importance in the clinical models.

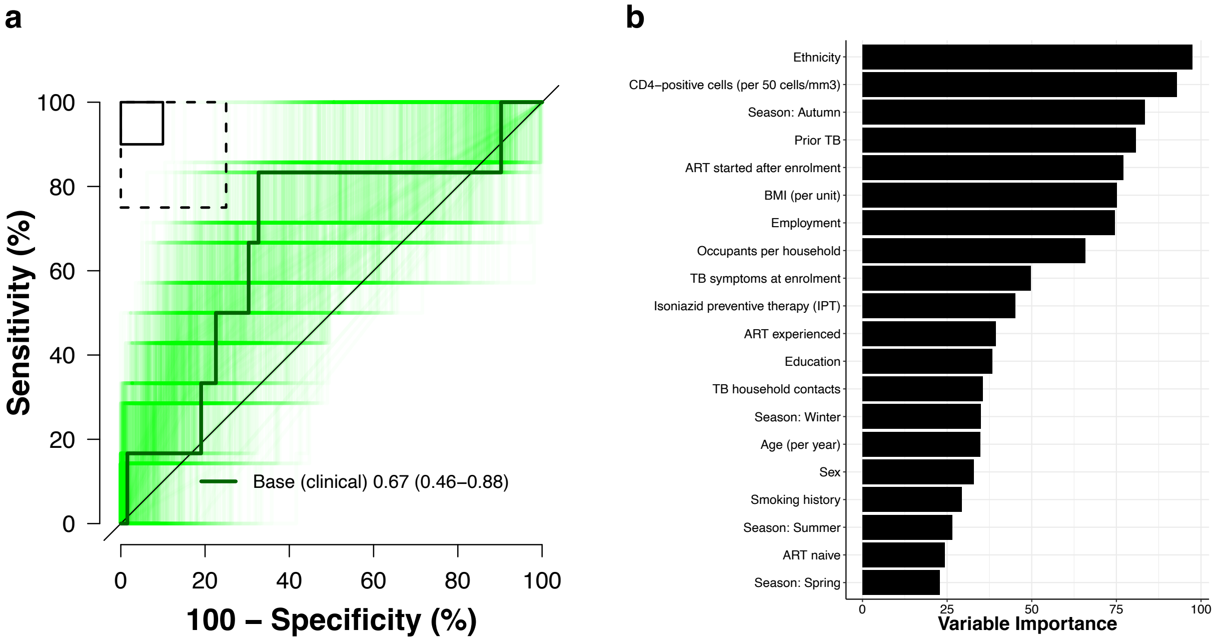

Supplementary Figure 3: Prognostic performance for incident TB through 15 months follow-up of LASSO regression clinical prediction models derived and tested in the CORTIS-HR cohort

(a) The receiver operating characteristic (ROC) curves depict test set prognostic performance (area under the curve, AUC, with 95% CI) of 1,000 LASSO regression clinical prediction models for incident TB diagnosed through 15 months follow-up on one or more liquid culture-positive or Xpert MTB/RIF-positive sputum samples. Each of the 1,000 green ROC curves depict performance in an individual test set. The mean cross-validated AUC is represented in dark green. The solid box depicts the optimal criteria (90% sensitivity and 90% specificity) and the dashed box depicts the minimal criteria (75% sensitivity and 75% specificity) set out in the WHO Target Product Profile for an incipient TB test.[12] (b) Relative feature importance in the clinical models.

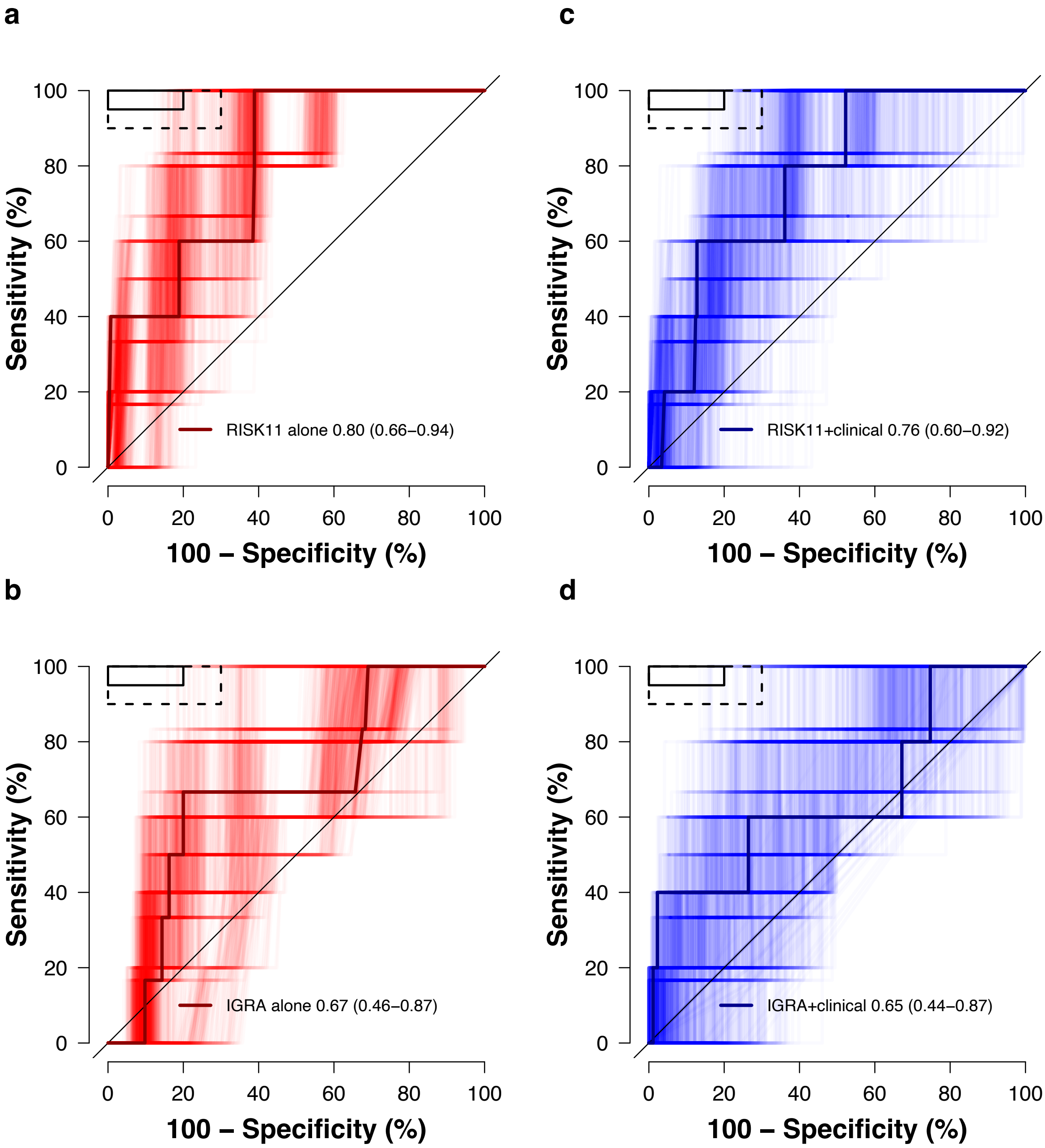

Supplementary Figure 4: Diagnostic performance of LASSO regression clinical prediction models incorporating RISK11 or the interferon-γ release assay derived and tested in the CORTIS-HR cohort

The receiver operating characteristic (ROC) curves depict test set diagnostic performance (area under the curve, AUC, with 95% CI) of (a) RISK11 alone and (b) the interferon-γ release assay (IGRA) alone, and 1,000 LASSO regression clinical prediction models incorporating (c) RISK11 or (d) IGRA, for prevalent TB diagnosed on one or more liquid culture-positive or Xpert MTB/RIF-positive sputum samples. Each of the 1,000 red or blue ROC curves depict performance in an individual test set. The mean cross-validated AUC is represented in dark red or dark blue. The solid box depicts the optimal criteria (95% sensitivity and 80% specificity) and the dashed box depicts the minimal criteria (90% sensitivity and 70% specificity) set out in the WHO Target Product Profile for a TB triage test.[11]

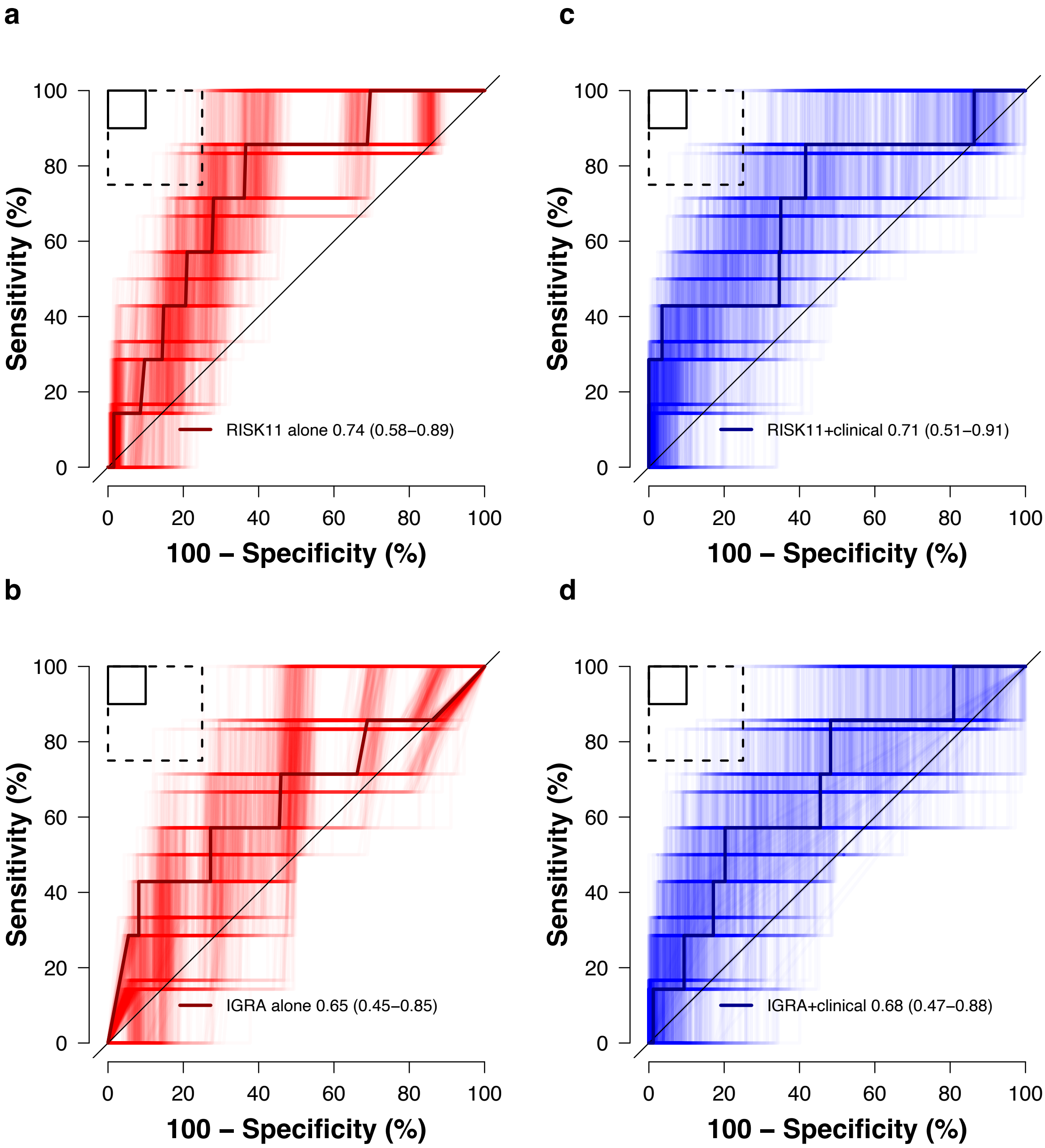

Supplementary Figure 5: Prognostic performance for incident TB through 15 months follow-up of LASSO regression clinical prediction models incorporating RISK11 or the interferon-γ release assay derived and tested in the CORTIS-HR cohort

The receiver operating characteristic (ROC) curves depict test set prognostic performance (area under the curve, AUC, with 95% CI) of (a) RISK11 alone and (b) the interferon-γ release assay (IGRA) alone, and 1,000 LASSO regression clinical prediction models incorporating (c) RISK11 or (d) IGRA, for incident TB diagnosed through 15 months follow-up on one or more liquid culture-positive or Xpert MTB/RIF-positive sputum samples. Each of the 1,000 red or blue ROC curves depict performance in an individual test set. The mean cross-validated AUC is represented in dark red or dark blue. The solid box depicts the optimal criteria (90% sensitivity and 90% specificity) and the dashed box depicts the minimal criteria (75% sensitivity and 75% specificity) set out in the WHO Target Product Profile for an incipient TB test.[12]

Supplementary Tables

Supplementary Table 1: Regression analyses examining factors associated with *Mycobacterium tuberculosis* sensitisation in people living with HIV

	Study cohort N = 851	IGRA-, n (%) or median (IQR) N=460	IGRA+, n (%) or median (IQR) N=391	Univariable logistic regression		Multivariable binomial LASSO regression with selective inference	
				**OR (95%CI)**	**p value**	**aOR (95%CI)**	**p value**
Sex							
Female	617 (72·5)	344 (74·8)	273 (69·8)	Reference		Reference	
Male	234 (27·5)	116 (25·2)	118 (30·2)	1·28 (0·95, 1·73)	0·11	1·11 (0·13, 1·25)	0·78
Age (years)	35 (29-42)	35 (29-42)	35 (29-42)	1·00 (0·98, 1·01)	0·86	-	-
Ethnicity							
Black African	716 (84·1)	400 (87·0)	316 (80·8)	Reference		Reference	
Mixed Ancestry	135 (15·9)	60 (13·0)	75 (19·2)	1·58 (1·09, 2·30)	**0·015**	1·10 (0·41, 1·28)	0·62
Site					**<0·001**‡		
Durban, KwaZulu-Natal	295 (34·7)	166 (36·1)	129 (33·0)	Reference		-	-
Klerksdorp, North West	160 (18·8)	69 (15·0)	91 (23·3)	1·70 (1·15, 2·51)	**0·008**	1·27 (1·00, 1·49) †	**0·024**
Rustenburg, North West	158 (18·6)	107 (23·3)	51 (13·0)	0·61 (0·41, 0·92)	**0·018**	0·86 (0·67, 1·06) †	0·069
Ravensmead, Western Cape	89 (10·5)	42 (9·1)	47 (12·0)	1·44 (0·90, 2·32)	0·13	-	-
Worcester, Western Cape	149 (17·5)	76 (16·5)	73 (18·7)	1·24 (0·83, 1·84)	0·29	-	-
Highest level of schooling							
Primary school or lower	100 (11·8)	49 (10·7)	51 (13·0)	Reference		Reference	
Secondary school or higher	751 (88·2)	411 (89·3)	340 (87·0)	0·79 (0·52, 1·21)	0·28	0·93 (0·82, 1·82)	0·58
Employment							
Employed	134 (15·7)	77 (16·7)	57 (14·6)	Reference		Reference	
Unemployed	717 (84·3)	383 (83·3)	334 (85·4)	1·18 (0·81, 1·72)	0·39	1·08 (0·38, 1·21)	0·68
Occupants per household	4 (3-6)	4 (3-7)	4 (3-6)	0·97 (0·93, 1·02)	0·25	0·89 (0·69, 1·28)	0·26
Smoking history							
No	522 (61·3)	309 (67·2)	213 (54·5)	Reference		Reference	
Yes	329 (38·7)	151 (32·8)	178 (45·5)	1·71 (1·30, 2·26)	**<0·001**	1·19 (0·96, 2·97)	**0·040**
Prior TB							
No	643 (75·6)	348 (75·7)	295 (75·4)	Reference			
Yes	208 (24·4)	112 (24·3)	96 (24·6)	1·01 (0·74, 1·38)	0·94	-	-
TB household contacts							
No	691 (81·2)	381 (82·8)	310 (79·3)	Reference		Reference	
Yes	160 (18·8)	79 (17·2)	81 (20·7)	1·26 (0·89, 1·78)	0·19	1·11 (0·76, 1·28)	0·31
Isoniazid preventive therapy at enrolment							
Not on therapy	805 (94·6)	433 (94·1)	372 (95·1)	Reference			
On therapy	46 (5·4)	27 (5·9)	19 (4·9)	0·82 (0·44, 1·49)	0·52	-	-
Antiretroviral therapy at enrolment					0·16‡		
Naïve	190 (22·3)	94 (20·4)	96 (24·6)	Reference		-	-
<6 months	114 (13·4)	57 (12·4)	57 (14·6)	0·98 (0·62, 1·56)	0·93	-	-
≥6 months	547 (64·3)	309 (67·2)	238 (60·9)	0·75 (0·54, 1·05)	0·095	0·85 (0·72, 1·06) †	0·070
Body-mass index (kg/m^2)	24·2 (20·6-31·2)	24·5 (20·1-31·6)	24·1 (20·9-30·5)	1·00 (0·98, 1·02)	0·90	1·14 (0·29, 1·29)	0·65
CD4-positive cell count (cells/mm^3)	530·0 (353·5-726·0)	485·5 (327·5-691·2)	567·0 (393·5-770·0)	1·05 (1·02, 1·07) *	**<0·001**	1·48 (1·12, 1·77) *	**0·006**
HIV plasma viral load							
<100 copies/mL	180 (44·1)	94 (43·1)	86 (45·3)	Reference		#	#
≥100 copies/mL	228 (55·9)	124 (56·9)	104 (54·7)	0·92 (0·62, 1·36)	0·66		
Not tested (missing)	443	242	201	-	-		
Season at enrolment					**0·013**‡		
Spring	204 (24·0)	95 (20·7)	109 (27·9)	Reference		1·02 (0·46, 1·22) †	0·72
Summer	220 (25·9)	131 (28·5)	89 (22·8)	0·59 (0·40, 0·87)	**0·008**	0·85 (0·57, 1·21) †	0·15
Autumn	191 (22·4)	114 (24·8)	77 (19·7)	0·59 (0·39, 0·88)	**0·009**	0·82 (0·56, 1·10) †	0·076
Winter	236 (27·7)	120 (26·1)	116 (29·7)	0·84 (0·58, 1·23)	0·37	-	-
TB symptoms at enrolment							
No	804 (94·5)	442 (96·1)	362 (92·6)	Reference		Reference	
Yes	47 (5·5)	18 (3·9)	29 (7·4)	1·97 (1·08, 3·66)	**0·028**	1·13 (0·86, 1·32)	0·17
TB status					**0·016**‡		
No TB	813 (95·5)	448 (97·4)	365 (93·4)	Reference		0·80 (0·69, 0·94) †	**0·006**
Prevalent TB	17 (2·0)	5 (1·1)	12 (3·1)	2·95 (1·08, 9·33)	**0·044**	-	-
Incident TB	21 (2·5)	7 (1·5)	14 (3·6)	2·45 (1·01, 6·54)	0·055	-	-

IQR, interquartile range. CI, confidence interval. OR, odds ratio. aOR, adjusted OR. IGRA, interferon-γ release assay. TB, tuberculosis. *Per 50 cells/mm^3. #Excluded from LASSO analysis. †Reference group includes all other levels of variable. ‡Likelihood-ratio test for significance of categorical variable.

Supplementary Table 2: Baseline characteristics of CORTIS-HR study cohort by clinical site in all enrolled participants

	Overall N=861	Durban N=300	Klerksdorp N=160	Rustenburg N=161	Ravensmead N=90	Worcester N=150
**Baseline characteristics at study enrolment**						
Female sex, n (%)	621 (72·1)	217 (72·3)	102 (63·7)	113 (70·2)	66 (73·3)	123 (82·0)
Median age, years (IQR)	35 (29-42)	35 (29-42)	32 (27-38)	34 (29-40)	38 (30-46)	38 (31-44)
Ethnicity, n (%)						
Black African	724 (84·1)	299 (99·7)	159 (99·4)	160 (99·4)	3 (3·3)	103 (68·7)
Mixed Ancestry	137 (15·9)	1 (0·3)	1 (0·6)	1 (0·6)	87 (96·7)	47 (31·3)
Median body-mass index, kg/m^2 (IQR)	24·2 (20·6-31·2)	25·9 (21·7-32·3)	22·6 (19·3-29·7)	23·8 (20·3-29·8)	20·7 (18·8-27·2)	26·2 (21·9-32·2)
Smoking history, n (%)	334 (38·8)	92 (30·7)	61 (38·1)	43 (26·7)	69 (76·7)	69 (46·0)
Prior tuberculosis, n (%)	212 (24·6)	73 (24·3)	31 (19·4)	25 (15·5)	32 (35·6)	51 (34·0)
Tuberculosis household contacts, n (%)	160 (18·6)	77 (25·7)	21 (13·1)	23 (14·3)	30 (33·3)	9 (6·0)
IGRA result, n (%)						
Not available	7 (0·8)	4 (1·3)	0	3 (1·9)	0	0
Negative	461 (53·5)	167 (55·7)	69 (43·1)	107 (66·5)	42 (46·7)	76 (50·7)
Positive	393 (45·6)	129 (43·0)	91 (56·9)	51 (31·7)	48 (53·3)	74 (49·3)
Isoniazid preventive therapy (IPT), n (%)						
On IPT at enrolment	47 (5·5)	10 (3·3)	11 (6·9)	15 (9·3)	8 (8·9)	3 (2·0)
Started IPT after enrolment	370 (43·0)	262 (87·3)	18 (11·2)	24 (14·9)	38 (42·2)	28 (18·7)
Did not take IPT during study	444 (51·6)	28 (9·3)	131 (81·9)	122 (75·8)	44 (48·9)	119 (79·3)
Antiretroviral therapy at enrolment, n (%)						
Naïve	193 (22·4)	57 (19·0)	48 (30·0)	40 (24·8)	12 (13·3)	36 (24·0)
<6 months	115 (13·4)	27 (9·0)	33 (20·6)	32 (19·9)	16 (17·8)	7 (4·7)
6-12 months	66 (7·7)	23 (7·7)	17 (10·6)	12 (7·5)	9 (10·0)	5 (3·3)
>12 months	487 (56·6)	193 (64·3)	62 (38·8)	77 (47·8)	53 (58·9)	102 (68·0)
Started antiretroviral therapy during study, n (%)	142/193 (73·6)	50/57 (87·7)	37/48 (77·1)	27/40 (67·5)	5/12 (41·7)	23/36 (63·9)
Median CD4-positive cells, $cells/mm^3$ (IQR)	529 (349·5-724·5)	544 (344·5-713·5)	532·5 (361·8-735)	545 (361-748)	515 (335-735)	495·5 (348-697)
Tuberculosis symptoms positive at enrolment, n (%)	51 (5·9)	21 (7·0)	1 (0·6)	3 (1·9)	18 (20·0)	8 (5·3)
**Tuberculosis endpoints (≥1 sputum sample positive)**						
Prevalent tuberculosis, n (%; 95% CI)	18 (2·1; 1·3-3·3)	7 (2·3; 1·1-4·7)	0	1 (0·6; 0·1-3·4)	0	10 (6·7; 3·7-11·8)
Incident tuberculosis, n (rate per 100 person-years; 95% CI)	21 (2·3; 1·3-3·2)	6 (1·8; 0·4-3·1)	1 (0·5; 0-1·5)	2 (1·2; 0-2·8)	5 (5·7; 0·7-10·3)	7 (4·3; 1·1-7·4)

IQR, inter-quartile range. IGRA, interferon-γ release assay. CI, confidence interval.

Supplementary Table 3: Regression analyses examining factors associated with *Mycobacterium tuberculosis* sensitisation in people living with HIV (sensitivity analysis with site removed from LASSO regression)

	Study cohort N = 851	IGRA-, n (%) or median (IQR) N=460	IGRA+, n (%) or median (IQR) N=391	Multivariable binomial LASSO regression with selective inference	
				aOR (95%CI)	p value
Sex					
Female	617 (72·5)	344 (74·8)	273 (69·8)	Reference	
Male	234 (27·5)	116 (25·2)	118 (30·2)	1·11 (0·25, 1·27)	0·71
Age (years)	35 (29-42)	35 (29-42)	35 (29-42)	-	-
Ethnicity					
Black African	716 (84·1)	400 (87·0)	316 (80·8)	Reference	
Mixed Ancestry	135 (15·9)	60 (13·0)	75 (19·2)	1·07 (0·37, 1·24)	0·69
Site				#	#
Durban, KwaZulu-Natal	295 (34·7)	166 (36·1)	129 (33·0)		
Klerksdorp, North West	160 (18·8)	69 (15·0)	91 (23·3)		
Rustenburg, North West	158 (18·6)	107 (23·3)	51 (13·0)		
Ravensmead, Western Cape	89 (10·5)	42 (9·1)	47 (12·0)		
Worcester, Western Cape	149 (17·5)	76 (16·5)	73 (18·7)		
Highest level of schooling					
Primary school or lower	100 (11·8)	49 (10·7)	51 (13·0)	Reference	
Secondary school or higher	751 (88·2)	411 (89·3)	340 (87·0)	0·95 (0·84, 2·23)	0·69
Employment					
Employed	134 (15·7)	77 (16·7)	57 (14·6)	Reference	
Unemployed	717 (84·3)	383 (83·3)	334 (85·4)	1·1 (0·61, 1·25)	0·45
Occupants per household	4 (3-6)	4 (3-7)	4 (3-6)	0·87 (0·71, 1·13)	0·13
Smoking history					
No	522 (61·3)	309 (67·2)	213 (54·5)	Reference	
Yes	329 (38·7)	151 (32·8)	178 (45·5)	1·22 (1·00, 2·32)	**0·024**
Prior TB					
No	643 (75·6)	348 (75·7)	295 (75·4)		
Yes	208 (24·4)	112 (24·3)	96 (24·6)	-	-
TB household contacts					
No	691 (81·2)	381 (82·8)	310 (79·3)	Reference	
Yes	160 (18·8)	79 (17·2)	81 (20·7)	1·11 (0·78, 1·27)	0·29
Isoniazid preventive therapy at enrolment					
Not on therapy	805 (94·6)	433 (94·1)	372 (95·1)		
On therapy	46 (5·4)	27 (5·9)	19 (4·9)	-	-
Antiretroviral therapy at enrolment					
Naïve	190 (22·3)	94 (20·4)	96 (24·6)	-	-
<6 months	114 (13·4)	57 (12·4)	57 (14·6)	-	-
≥6 months	547 (64·3)	309 (67·2)	238 (60·9)	0·84 (0·72, 1·02) †	**0·036**
Body-mass index (kg/m^2)	24·2 (20·6-31·2)	24·5 (20·1-31·6)	24·1 (20·9-30·5)	1·13 (0·43, 1·29)	0·57
CD4-positive cell count (cells/mm^3)	530·0 (353·5-726·0)	485·5 (327·5-691·2)	567·0 (393·5-770·0)	1·47 (1·19, 1·72) *	**0·001**
HIV plasma viral load				#	#
<100 copies/mL	180 (44·1)	94 (43·1)	86 (45·3)		
≥100 copies/mL	228 (55·9)	124 (56·9)	104 (54·7)		
Not tested (missing)	443	242	201		
Season at enrolment					
Spring	204 (24·0)	95 (20·7)	109 (27·9)	1·02 (0·40, 1·20) †	0·76
Summer	220 (25·9)	131 (28·5)	89 (22·8)	0·86 (0·55, 1·17) †	0·13
Autumn	191 (22·4)	114 (24·8)	77 (19·7)	0·84 (0·54, 1·11)†	0·087
Winter	236 (27·7)	120 (26·1)	116 (29·7)	-	-
TB symptoms at enrolment					
No	804 (94·5)	442 (96·1)	362 (92·6)	Reference	
Yes	47 (5·5)	18 (3·9)	29 (7·4)	1·12 (0·86, 1·34)	0·18
TB status					
No TB	813 (95·5)	448 (97·4)	365 (93·4)	0·81 (0·69, 0·95) †	**0·008**
Prevalent TB	17 (2·0)	5 (1·1)	12 (3·1)	-	-
Incident TB	21 (2·5)	7 (1·5)	14 (3·6)	-	-

IQR, interquartile range. CI, confidence interval. OR, odds ratio. aOR, adjusted OR. IGRA, interferon-γ release assay. TB, tuberculosis. *Per 50 cells/mm^3. #Excluded from LASSO analysis. †Reference group includes all other levels of variable.

Supplementary Table 4: Regression analyses examining factors associated with prevalent TB disease in people living with HIV (sensitivity analysis with site removed from LASSO regression)

	No prevalent TB, n (%) or median (IQR)	Prevalent TB, n (%) or median (IQR)	Multivariable binomial LASSO regression with selective inference	
	N=834	N=17	aOR (95%CI)	p value
Sex				
Female	608 (72·9)	9 (52·9)		
Male	226 (27·1)	8 (47·1)	-	-
Age	35 (29-42)	36 (33-42)	1·38 (0·09, 9·12)	0·45
Ethnicity				
Black African	704 (84·4)	12 (70·6)	Reference	
Mixed Ancestry	130 (15·6)	5 (29·4)	1·20 (0·13, >1000)	0·18
Site				
Durban, KwaZulu-Natal	288 (34·5)	7 (41·2)		
Klerksdorp, North West	160 (19·2)	0 (0·0)	#	#
Rustenburg, North West	158 (18·9)	0 (0·0)		
Ravensmead, Western Cape	89 (10·7)	0 (0·0)		
Worcester, Western Cape	139 (16·7)	10 (58·8)		
Highest level of schooling				
Primary school or lower	100 (12·0)	0 (0·0)	Reference	
Secondary school or higher	734 (88·0)	17 (100·0)	2·15 (0·48, 5·85)	0·14
Employment				
Employed	131 (15·7)	3 (17·6)		
Unemployed	703 (84·3)	14 (82·4)	-	-
Occupants per household	4 (3-6)	4 (1-6)	0·80 (0·51, 345·73)	0·80
Smoking history				
No	513 (61·5)	9 (52·9)		
Yes	321 (38·5)	8 (47·1)	-	-
Prior TB				
No	630 (75·5)	13 (76·5)		
Yes	204 (24·5)	4 (23·5)	-	-
TB household contacts				
No	674 (80·8)	17 (100·0)	Reference	
Yes	160 (19·2)	0 (0·0)	0·36 (0·03, 1·13)	**0·035**
Isoniazid preventive therapy at enrolment				
Not on therapy	788 (94·5)	17 (100·0)	Reference	
On therapy	46 (5·5)	0 (0·0)	0·78 (0·25, 169·67)	0·72
Antiretroviral therapy at enrolment				
Naïve	183 (21·9)	7 (41·2)	1·21 (0·01, 2·04) †	0·77
<6 months	112 (13·4)	2 (11·8)	-	-
≥6 months	539 (64·6)	8 (47·1)	-	-
Body-mass index (kg/m^2)	24·3 (20·6-31·3)	21·3 (18·7-24·0)	0·57 (0·05, 7·42)	0·29
CD4-positive cell count (cells/mm^3)	530·5 (354·2-731·0)	393·0 (248·0-591·0)	0·54 (0·00, 1·16) *	**0·040**
HIV plasma viral load				
<100 copies/mL	179 (45·1)	1 (9·1)	#	#
≥100 copies/mL	218 (54·9)	10 (90·9)		
Not tested (missing)	437	6		
Season at enrolment				
Spring	196 (23·5)	8 (47·1)	1·47 (1·23, >1000) †	**0·014**
Summer	218 (26·1)	2 (11·8)	0·83 (0·69, >1000) †	0·98
Autumn	188 (22·5)	3 (17·6)	-	-
Winter	232 (27·8)	4 (23·5)	-	-
TB symptoms at enrolment				
No	789 (94·6)	15 (88·2)	Reference	
Yes	45 (5·4)	2 (11·8)	1·09 (0·00, 1·02)	0·97
IGRA status				
Negative	455 (54·6)	5 (29·4)	Reference	
Positive	379 (45·4)	12 (70·6)	1·76 (0·89, 60·02)	**0·038**

IQR, interquartile range. CI, confidence interval. OR, odds ratio. aOR, adjusted OR. IGRA, interferon-γ release assay. TB, tuberculosis. NA, not applicable. *Per 50 cells/mm^3. #Excluded from LASSO analysis. †Reference group includes all other levels of variable.

Supplementary Table 5: Regression analyses examining baseline factors associated with progression to incident TB disease within 15 months in people living with HIV (sensitivity analysis with site removed from LASSO regression)

	No incident TB, n (%) or median (IQR)	Incident TB, n (%) or median (IQR)	Multivariable Cox LASSO regression with selective inference	
	**N=808**	**N=21**	**aHR (95%CI)**	**p value**
Sex				
Female	590 (73·0)	15 (71·4)	Reference	
Male	218 (27·0)	6 (28·6)	0·71 (0·75, >1000)	0·95
Age	35 (29-42)	33 (30-40)	0·75 (0·03, 10·16)	0·36
Ethnicity				
Black African	689 (85·3)	11 (52·4)	Reference	
Mixed Ancestry	119 (14·7)	10 (47·6)	1·56 (1·22, 209·44)	**0·004**
Site				
Durban, KwaZulu-Natal	281 (34·8)	6 (28·6)		
Klerksdorp, North West	158 (19·6)	1 (4·8)	#	#
Rustenburg, North West	154 (19·1)	2 (9·5)		
Ravensmead, Western Cape	84 (10·4)	5 (23·8)		
Worcester, Western Cape	131 (16·2)	7 (33·3)		
Highest level of schooling				
Primary school or lower	95 (11·8)	5 (23·8)	Reference	
Secondary school or higher	713 (88·2)	16 (76·2)	0·84 (0·60, 20·04)	0·73
Employment				
Employed	130 (16·1)	1 (4·8)	Reference	
Unemployed	678 (83·9)	20 (95·2)	1·78 (0·52, 3·49)	0·18
Occupants per household	4 (3-6)	4 (3-8)	1·20 (0·07, 2·70)	0·64
Smoking history				
No	501 (62·0)	9 (42·9)		
Yes	307 (38·0)	12 (57·1)	NA	NA
Prior TB				
No	614 (76·0)	12 (57·1)	Reference	
Yes	194 (24·0)	9 (42·9)	1·75 (0·13, 2·44)	0·44
TB household contacts				
No	654 (80·9)	16 (76·2)		
Yes	154 (19·1)	5 (23·8)	NA	NA
Isoniazid preventive therapy during study conduct				
Did not receive therapy	409 (50·6)	13 (61·9)	Reference	
Received therapy	399 (49·4)	8 (38·1)	0·72 (0·60, 224·52)	0·81
Antiretroviral therapy (ART) during study conduct				
ART experienced	635 (78·6)	12 (57·1)	-	
ART naïve	42 (5·2)	1 (4·8)	-	
ART started after enrolment	131 (16·2)	8 (38·1)	1·55 (0·11, 2·08) †	0·52
Body-mass index (kg/m^2)	24·5 (20·7-31·5)	19·9 (18·5-24·9)	0·52 (0·39, >1000)	0·84
CD4-positive cell count (cells/mm^3)	533·5 (360·8-734·2)	291·0 (145·0-573·0)	0·44 (0·28, 12·54) *	0·39
HIV plasma viral load				
<100 copies/mL	175 (46·1)	3 (20·0)	#	#
≥100 copies/mL	205 (53·9)	12 (80·0)		
Not tested (missing)	428	6		
Season at enrolment				
Spring	190 (23·5)	6 (28·6)	-	-
Summer	210 (26·0)	5 (23·8)	-	-
Autumn	187 (23·1)	1 (4·8)	0·44 (0·24, 1·40) †	0·089
Winter	221 (27·4)	9 (42·9)	-	-
TB symptoms at enrolment				
No	764 (94·6)	20 (95·2)		
Yes	44 (5·4)	1 (4·8)	-	-
IGRA status				
Negative	446 (55·2)	7 (33·3)	Reference	
Positive	362 (44·8)	14 (66·7)	1·51 (0·03, 2·13)	0·64

IQR, interquartile range. CI, confidence interval. HR, hazard ratio. aHR, adjusted hazard ratio. IGRA, interferon-γ release assay. TB, tuberculosis. *Per 50 cells/mm^3. #Excluded from LASSO analysis. †Reference group includes all other levels of variable.

Supplementary Table 6: Regression analyses examining factors associated with cumulative prevalent and incident TB disease in people living with HIV

	No prevalent or incident TB, n (%) or median (IQR)	Cumulative prevalent & incident TB, n (%) or median (IQR)	Univariable logistic regression		Multivariable binomial LASSO regression with selective inference	
	N=813	N=38	OR (95%CI)	p value	aOR (95%CI)	p value
Sex						
Female	593 (72·9)	24 (63·2)	Reference			
Male	220 (27·1)	14 (36·8)	1·57 (0·78, 3·06)	0·19	-	-
Age	35·0 (29·0-42·0)	34·0 (31·0-41·5)	1·00 (0·96, 1·04)	0·96	-	-
Ethnicity						
Black African	693 (85·2)	23 (60·5)	Reference		Reference	
Mixed Ancestry	120 (14·8)	15 (39·5)	3·77 (1·88, 7·36)	**<0·001**	1·25 (0·05, 5·80)	0·58
Site				**<0·001‡**	1·43 (0·00, 32·88) † 0·62 (0·00, 14·10) †	
Durban, KwaZulu-Natal	282 (34·7)	13 (34·2)	Reference		0·89 (0·00, 53·18)	0·65
Klerksdorp, North West	159 (19·6)	1 (2·6)	0·14 (0·01, 0·69)	0·056	†	0·23
Rustenburg, North West	156 (19·2)	2 (5·3)	0·28 (0·04, 1·02)	0·095	-	0·40
Ravensmead, Western Cape	84 (10·3)	5 (13·2)	1·29 (0·40, 3·53)	0·64	1·75 (0·03, 11·03)	-
Worcester, Western Cape	132 (16·2)	17 (44·7)	2·79 (1·32, 6·03)	**0·007**	†	0·50
Highest level of schooling						
Primary school or lower	95 (11·7)	5 (13·2)	Reference			
Secondary school or higher	718 (88·3)	33 (86·8)	0·87 (0·36, 2·60)	0·78	-	-
Employment						
Employed	130 (16·0)	4 (10·5)	Reference		Reference	
Unemployed	683 (84·0)	34 (89·5)	1·62 (0·63, 5·49)	0·37	1·28 (0·29, 1·75)	0·50
Occupants per household	4·0 (3·0-6·0)	4·0 (3·0-6·0)	1·01 (0·90, 1·12)	0·82	-	-
Smoking history						
No	504 (62·0)	18 (47·4)	Reference			
Yes	309 (38·0)	20 (52·6)	1·81 (0·94, 3·51)	0·074	-	-
Prior TB						
No	618 (76·0)	25 (65·8)	Reference		Reference	
Yes	195 (24·0)	13 (34·2)	1·65 (0·80, 3·23)	0·16	1·17 (0·04, 1·52)	0·77
TB household contacts						
No	658 (80·9)	33 (86·8)	Reference			
Yes	155 (19·1)	5 (13·2)	0·64 (0·22, 1·53)	0·37	-	-
Isoniazid preventive therapy at enrolment						
Not on therapy	767 (94·3)	38 (100·0)	NA	NA	Reference	
On therapy	46 (5·7)	0 (0·0)			0·76 (0·50, 12·73)	0·63
Antiretroviral therapy at enrolment				**0·020‡**		
Naïve	174 (21·4)	16 (42·1)	Reference		1·22 (0·55, 3·20) †	0·27
<6 months	110 (13·5)	4 (10·5)	0·40 (0·11, 1·11)	0·10	-	-
≥6 months	529 (65·1)	18 (47·4)	0·37 (0·18, 0·75)	**0·005**	-	-
Body-mass index (kg/m^2)	24·5 (20·7-31·4)	21·0 (18·6-24·7)	0·89 (0·83, 0·95)	**<0·001**	0·47 (0·28, 0·78)	**0·003**
CD4-positive cell count (cells/mm^3)	533·0 (360·0-733·0)	357·5 (158·8-588·0)	0·88 (0·81, 0·94) *	**<0·001**	0·51 (0·34, 0·81)	**0·004**
HIV plasma viral load						
<100 copies/mL	176 (46·1)	4 (15·4)	Reference		#	#
≥100 copies/mL	206 (53·9)	22 (84·6)	4·7 (1·76, 16·3)	**0·005**		
Not tested (missing)	431	12	NA	NA		
Season at enrolment				0·072‡		
Spring	190 (23·4)	14 (36·8)	Reference		1·09 (0·02, 1·40) †	0·85
Summer	213 (26·2)	7 (18·4)	0·45 (0·17, 1·10)	0·088	-	-
Autumn	187 (23·0)	4 (10·5)	0·29 (0·08, 0·83)	**0·032**	0·75 (0·16, 2·14) †	0·23
Winter	223 (27·4)	13 (34·2)	0·79 (0·36, 1·73)	0·56	-	-
TB symptoms at enrolment						
No	769 (94·6)	35 (92·1)	Reference			
Yes	44 (5·4)	3 (7·9)	1·50 (0·35, 4·38)	0·52	-	-
IGRA status						
Negative	448 (55·1)	12 (31·6)	Reference		Reference	
Positive	365 (44·9)	26 (68·4)	2·66 (1·35, 5·53)	**0·006**	1·58 (1·01, 2·59)	**0·023**

IQR, interquartile range. CI, confidence interval. OR, odds ratio. aOR, adjusted OR. IGRA, interferon-γ release assay. TB, tuberculosis. NA, not applicable. *Per 50 cells/mm^3. #Excluded from LASSO analysis. †Reference group includes all other levels of variable. ‡Likelihood-ratio test for significance of categorical variable.

References

1. Dorai-Raj S. binom: Binomial Confidence Intervals For Several Parameterizations. R package version 1.1-1. 2014. https://CRAN.R-project.org/package=binom (accessed October 01, 2021).
2. Aalen O. Nonparametric Inference for a Family of Counting Processes. *Annals of Statistics.* 1978; **6**(4): 701-26. doi: DOI 10.1214/aos/1176344247.
3. Friedman J, Hastie T, Tibshirani R, et al. Lasso and Elastic-Net Regularized Generalized Linear Models (Version 4.1-2). 2021. https://cran.r-project.org/web/packages/glmnet/glmnet.pdf (accessed July 22, 2021).
4. Breiman L, Spector P. Submodel Selection and Evaluation in Regression - the X-Random Case. *International Statistical Review.* 1992; **60**(3): 291-319. doi: Doi 10.2307/1403680.
5. Baik Y, Rickman HM, Hanrahan CF, et al. A clinical score for identifying active tuberculosis while awaiting microbiological results: Development and validation of a multivariable prediction model in sub-Saharan Africa. *PLoS Med.* 2020; **17**(11): e1003420. doi: 10.1371/journal.pmed.1003420.
6. Hanifa Y, Fielding KL, Chihota VN, et al. A clinical scoring system to prioritise investigation for tuberculosis among adults attending HIV clinics in South Africa. *PLoS ONE.* 2017; **12**(8): e0181519. doi: 10.1371/journal.pone.0181519.
7. Gupta RK, Calderwood CJ, Yavlinsky A, et al. Discovery and validation of a personalized risk predictor for incident tuberculosis in low transmission settings. *Nat Med.* 2020; **26**(12): 1941-9. doi: 10.1038/s41591-020-1076-0.
8. Robin X, Turck N, Hainard A, et al. pROC: an open-source package for R and S+ to analyze and compare ROC curves. *BMC Bioinformatics.* 2011; **12**: 77. doi: 10.1186/1471-2105-12-77.
9. DeLong ER, DeLong DM, Clarke-Pearson DL. Comparing the areas under two or more correlated receiver operating characteristic curves: a nonparametric approach. *Biometrics.* 1988; **44**(3): 837-45. doi: 10.2307/2531595.
10. Carpenter J, Bithell J. Bootstrap confidence intervals: when, which, what? A practical guide for medical statisticians. *Stat Med.* 2000; **19**(9): 1141-64. doi: 10.1002/(sici)1097-0258(20000515)19:9<1141::Aid-sim479>3.0.Co;2-f.
11. WHO. High-priority target product profiles for new tuberculosis diagnostics: report of a consensus meeting. Geneva: World Health Organization. 2014. https://apps.who.int/iris/handle/10665/135617 (accessed May 22, 2020).
12. WHO. Consensus Meeting Report: Development of a Target Product Profile (TPP) and a framework for evaluation for a test for predicting progression from tuberculosis infection to active disease. Geneva: World Health Organization. 2017. http://apps.who.int/iris/handle/10665/259176 (accessed May 22, 2020).
